# Mental Health Impacts of Climate Change Among Vulnerable Populations Globally: An Integrative Review

**DOI:** 10.5334/aogh.4105

**Published:** 2023-10-06

**Authors:** Bradley Patrick White, Suellen Breakey, Margaret J. Brown, Jenny Rand Smith, Amanda Tarbet, Patrice K. Nicholas, Ana M. Viamonte Ros

**Affiliations:** 1 MGH Institute of Health Professions School of Nursing, 36 1st Avenue, Boston, MA 02129, US; 2 SFPE Foundation, 9711 Washingtonian Blvd, Gaithersburg, MD 20878, US; 3 Center for Climate Change, Climate Justice, and Health, MGH Institute of Health Professions School of Nursing, 36 1@st Avenue, Boston, MA 02129, US; 4 Florida International University, Herbert Wertheim School of Medicine, Miami, Florida, US

**Keywords:** mental health, climate change and health, anxiety, post-traumatic stress disorder, solastalgia, ecoanxiety

## Abstract

**Background:**

Climate change has been shown to be directly linked to multiple physiological sequelae and to impact health consequences. However, the impact of climate change on mental health globally, particularly among vulnerable populations, is less well understood.

**Objective:**

To explore the mental health impacts of climate change in vulnerable populations globally.

**Methods:**

We performed an integrative literature review to identify published articles that addressed the research question: *What are the mental health impacts of climate change among vulnerable populations globally?* The Vulnerable Populations Conceptual Model served as a theoretical model during the review process and data synthesis.

**Findings/Results:**

One hundred and four articles were selected for inclusion in this review after a comprehensive review of 1828 manuscripts. Articles were diverse in scope and populations addressed. Land-vulnerable persons (either due to occupation or geographic location), Indigenous persons, children, older adults, and climate migrants were among the vulnerable populations whose mental health was most impacted by climate change. The most prevalent mental health responses to climate change included solastalgia, suicidality, depression, anxiety/eco-anxiety, PTSD, substance use, insomnia, and behavioral disturbance.

**Conclusions:**

Mental health professionals including physicians, nurses, physician assistants and other healthcare providers have the opportunity to mitigate the mental health impacts of climate change among vulnerable populations through assessment, preventative education and care. An inclusive and trauma-informed response to climate-related disasters, use of validated measures of mental health, and a long-term therapeutic relationship that extends beyond the immediate consequences of climate change-related events are approaches to successful mental health care in a climate-changing world.

## 1. Introduction

Climate change is the leading public health threat of the 21^st^ century and associated with deleterious health consequences. Our changing climate is due primarily to human activities or anthropogenic causes which have increased the average global temperature by 0.5 degrees Celsius with projections for the year 2100 indicating that average global temperature will rise by 2.4 to 5.8 degrees Celsius [1,2]. Notable physical health consequences include heat-related illness related to persistent rising temperatures, an increase in physical trauma and deaths due to extreme weather events, air quality impacts, vector-borne and water-related illnesses, and health impacts such as undernutrition, malnutrition, and obesity related to changes in food safety and distribution as well as decreases in micronutrients resulting from higher levels of atmospheric carbon dioxide [3]. The negative effects of climate change also impact mental health and well-being, but these consequences are less well-understood and understudied. Those particularly vulnerable to the negative physical and mental health impacts of climate change include those with low socioeconomic status, children, older adults, pregnant women, certain communities of color, Indigenous peoples, vulnerable occupational groups, people with disabilities and those with preexisting or chronic medical conditions [3]. The purpose of this integrative review is to examine how climate change impacts the mental health of vulnerable populations.

### 1.1 Background

Increasing ambient temperatures are linked with mental health consequences including increased rates of aggression and suicide, as well as conflict, violence, and migration [4,5]. Mental health consequences due to heat stress are increasingly common in most areas of the world, disproportionately affect vulnerable populations, and are most prevalent in certain occupational groups (e.g., farmworkers, miners, construction workers, factory workers) who are exposed to high temperatures in the workplace. Heat waves are associated with physical health consequences such as heat stress and heat stroke, as well as Mesoamerican nephropathy (chronic renal failure resulting from lack of access to adequate hydration and heat breaks in occupational workers). Recent studies suggest that heat waves and heat stress are associated with psychiatric sequelae including post-traumatic stress disorders, mood disorders, anxiety disorders, and dementia. A recent study in Thailand found that psychological stress was as negative an outcome as the physical health consequences of exposure to high ambient temperatures [6]. An Australian study found an association between heat waves increased rates of hospital admissions for mental disorders [7]. Aggressive behaviors, conflict, and violence are known to be higher in high temperature environments, and suicide is linked with increases in temperature and heat stress [8].

Recent reviews of mental health effects of climate change provided an analysis and framework for understanding how climate change affects mental health [3,4]. Kumar Padhy et al.’s [4] framework addressed ambient temperature and effects on mental health; psychological consequences due to climate-related disasters, drought and farmer suicide; economic loss due to climate change and the effects on mental health, migration and acculturation stress; and mental health associations with physical illnesses. Crimmins et al. [3] developed a scientific assessment of mental health and well-being related to climate change that yielded key findings. Key finding one addressed that exposure to disasters results in mental health consequences. The second key finding is that specific groups of people are at higher risk for distress and other adverse mental health consequences from exposure to climate-related or weather-related disasters Crimmins et al. [3] note that these groups include infants and children, women (particularly pregnant and postpartum women), people with pre-existing mental illness, economically disadvantaged, homeless, and first responders. The third key finding is that climate change threats result in mental health consequences and social impacts; these adverse mental health outcomes and social impacts from the threat of climate change, the perceived direct experience of climate change, and changes in one’s environment contribute to mental health sequelae. The fourth key finding is that extreme heat increases risks for people with mental illness. Further those with pre-existing mental health challenges are at higher risk of poor physical and mental health due to extreme heat and may lack the ability to limit heat exposure (lack of air conditioning, exposure to outside environment). In particular, Crimmins et al. [3] note that the elderly and those taking prescription medications may have impaired ability to regulate temperature. This conceptual framework is further explicated as a framework with application to mental health and climate change later in this paper.

### 1.2 The Emerging Literature on Solastalgia

Recent literature has focused on the issues related to climate anxiety, eco-anxiety, climate grief, and the recently new concept of solastalgia. These represent an emerging area of climate change and its intersection with mental health. Solastalgia refers to distress caused by the transformation, deterioration, and degradation of one’s environment with relevance to the *environment-health-place nexus* [9]. Knight [10] discussed the complex issues of climate anxiety and eco-anxiety and the intersection of anxiety and or/depression linked with climate change, noting that no epidemiological data exist to support how commonly distress and anxiety occur. A recent Gallup poll reported that 54 percent of those aged 18 to 34 years, 38 percent of those 35 to 54 years, and 44 percent of those 55 or older worry “a great deal” about climate change [11]. This supports the findings of the Yale School of Public Health and George Mason University’s national survey entitled *The Six Americas*, a survey conducted every four years to determine survey participants’ beliefs about climate change [12]. Their framework suggests that climate change communication should be viewed through the lens of *Six Americas*—a national survey that categorized people regarding their beliefs about climate change from those who are *Dismissive, Doubtful, Disengaged, Cautious, Concerned*, or *Alarmed*.

In their integrative review of the literature, Galway et al. [9] discussed solastalgia related to individuals and communities who are witnessing climate change and the associated environmental degradation that impacts physical health sequelae, as well as mental health consequences. Solastalgia is characterized as having often overlapping emotional, mental, and spiritual dimensions [9,40,50,96,131] Galway et al. [9] found that sources of environmental change causing solastalgia include extreme weather events/natural disasters (e.g., floods, droughts, hurricanes); prolonged environmental transformation; land clearing/deforestation, resource extraction/development (e.g., mining), gentrification/changing the built environment, displacement or appropriation of land/political violence/war; rapid industrial development; and climate change as a unique source of environmental change. Climate migration is exacerbated in the setting of violence, conflict, and war, and is well established as a unique contributor to mental health challenges [13].

### 1.3 Frameworks with Application to Mental Health Consequences of Climate Change

The Vulnerable Populations Conceptual Model (VPCM), developed by Flaskerud and Winslow [14]supports the interconnected relationship of three aspects that have relevance for climate change and mental health: *resources available, relative risk*, and *health status*. Through the lens of this model, *resources available* refers to both socioeconomic and environmental resources; *relative risk* refers to likelihood of exposure to a variety of well-documented risk factors; and *health status* refers to “age- and gender-specific morbidity and mortality [14].”

When applying the VPCM to climate change, *relative risk* can be conceptualized as risk of exposure to climate-related stressors compared to individuals in a healthy climate zone. *Resources available* can be conceptualized as access to clean water, clean air, stable temperatures, finances, and social support. *Health status* can be conceptualized as impact of climate-related exposure on morbidity and mortality data. Persons with pre-existing mental illness are classified as vulnerable populations [15] and are disproportionately affected by climate-related stressors [16]. An inter-connectedness between exposure to climate-related stressors, decreased availability of and access to resources, and decreased health status exists which further perpetuates resource availability and risk exposure [14]. Albarrán and Nyamathi [17] offer evidence of the relevance of this model for vulnerable populations across culture, socioeconomics, and migrant status, thus attesting to the model’s relevance in mental health sequelae of climate change.

**Figure 1:**
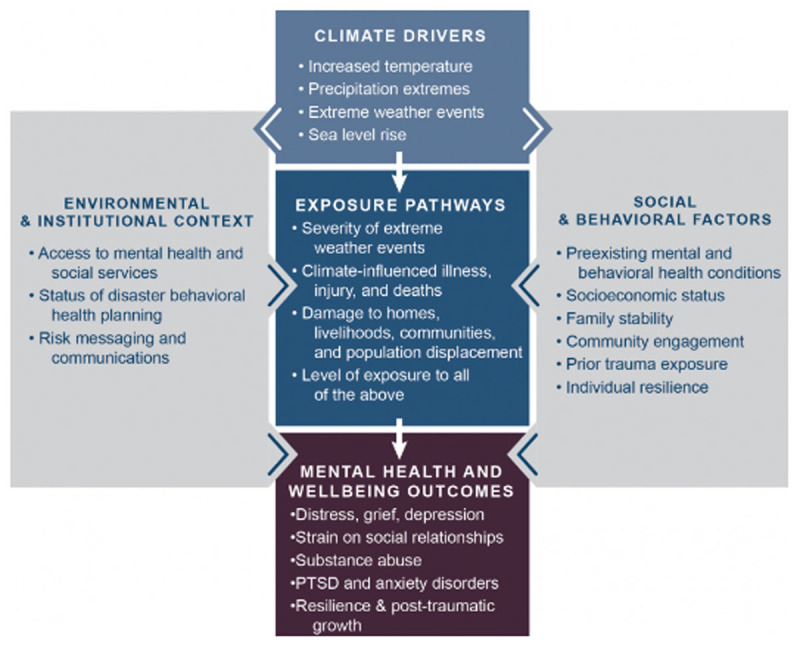
Climate Change and Mental Health and Wellness Conceptual Framework [3].

The Climate Change and Mental Health and Wellness Model (CCMHWM) was developed by Crimmins et al. [3] and published by the US Global Change Research Program. It identified key pathways by which humans are exposed to health threats from climate drivers, and possible deleterious outcomes to mental health and well-being. As described previously, the framework identified climate drivers, exposure pathways, and their impact on mental health and well-being outcomes. Climate drivers include increased temperature; precipitation extremes; extreme weather events; and sea level rise. Exposure pathways include severity of extreme weather events; climate-influenced illness, injury, and death; damage to homes, livelihoods, communities, and population displacement; and level of exposure to all of the above. These climate drivers and exposure pathways then impact mental health and well-being outcomes which may result in distress, grief, and depression; strain on social relationships; substance abuse; post-traumatic stress disorder and anxiety disorders; or resilience and post-traumatic growth (a potentially positive outcome). Social and behavioral factors that may influence health outcomes and vulnerability include pre-existing mental and behavioral health conditions, socioeconomic status, family stability, community engagement, prior trauma exposure, and individual resilience. Within the environmental and institutional context, access to mental health and social service resources; status of disaster behavioral health planning and risk messaging and communications influence mental health and well-being outcomes (Figure 1).

In this review, we explored these aspects of mental health consequences of climate change and sought to expand the understanding within a framework of mitigation, adaptation, and resilience in our climate-changing world.

## 2. Methods

Our integrative review aimed to explicate existing research and scholarly articles specifically examining mental health consequences of climate change in vulnerable populations. To develop and plan our approach to this integrative review, we referred to the approaches to conducting integrative reviews suggested by Whittemore and Knafl [18,19,20,22]. We searched several databases: MEDLINE using the Ovid platform (specifically Epub Ahead of Print, In-Process & Other Non-Indexed Citations, Ovid MEDLINE(R) Daily, Ovid MEDLINE and Versions(R) 1946 to Present), CINAHL Complete on Ebscohost, APA PsycInfo on Ovid, the Cochrane Database of Systematic Reviews on Ovid, Scopus, PTSD pubs, and Google Scholar on January 16, 2020. For Google Scholar, specifically only the first 200 search results were reviewed. Search terms and subject headings fell into one of three categories: a vulnerable population, aspects of mental health, or climate change and associated phenomena. A health sciences librarian developed the search query with input from the rest of the research team. For a complete list of search terms and modifiers used in Ovid MEDLINE (Table 1). The Ovid MEDLINE search query was adapted for all of the other databases. The search results were limited to a publication date of 1992 to 2020 and English language publications. Subsequently, we added search results from 2020 to 2022 to have an up-to-date review of current literature.

**Table 1:** Ovid MEDLINE Search Terms.


POPULATION	CONCEPT	CONTEXT

Vulnerable populations/	Mental health	*Climate Change/

vulnerable populations	Mental Health/	*Global Warming/

Indigenous	Solastalgia	Climate change

exp american native continental ancestry group/	Psychoterratic	Global warming

oceanic ancestry group/	Climate anxiety	Sea Level Rise

Health Services, Indigenous/	Eco-anxiety	Environmental change

Communities of color	Environmental distress	Greenhouse effect

minorities	Climate trauma	

Minority Groups/	exp Stress Disorders, Traumatic/	

Minority Health/	PTSD	

Homeless	Post-traumatic stress disorder	

exp Homeless Persons/	Anxiety	

Elderly	Anxiety/	

Aged/	Depression	

Health Services for the Aged/	Depression/	

Income inequality	exp Depressive Disorder/	

Poverty	Grief	

exp Poverty/	exp Grief/	

Children	Grieving	

exp Child/	Environmentally induced distress	

exp Infant/	Mental well-being	

Adolescent/	Stress	

Pregnant women	Stress, Psychological/	

Pregnant Women/	Resilience	

People with chronic illnesses	Resiliency	

Non-native speakers	Resilience, Psychological/	

Migrants		

Migrant workers		

“Transients and Migrants”[Mesh]		

Refugees		

Refugees/		

Immigrants		

exp Emigrants and Immigrants/		

Farmworkers		

LGBTQ		

exp Sexual and Gender Minorities/		

Construction workers		

Miners		

Miners/		

Women		

Women/		

People with disabilities		

exp Disabled Persons/		

Health Services for Persons with Disabilities/		

People with mental illness		

Global South		

Developing countries		

Developing Countries/		

Low income		

(“people with” adj3 (“chronic illness” or “chronic illnesses” or “mental illness” or “mental illnesses” or disabilities or “chronic disease” or “chronic diseases”))		


All of the authors participated in the title and abstract screening stage. To reduce bias, we screened articles using Covidence [21], a review management tool that reduces bias in the review process by facilitating independent screening. We then screened the full text of the articles that passed the initial screening stage for inclusion in the review. Each article was screened by two reviewers working independently. Consensus was reached via face-to-face discussion.

In addition to answering the research question, articles must also have met the following eligibility criteria for inclusion: original research, case study, theoretical/conceptual framework, policy analysis, expert analysis, commentary, integrative review or systematic review, any geographic location, published after 1992, and human subjects. The decision to include scholarly literature that was not research was based on Whittemore and Knafl’s [22] description of the purpose of an integrative review, which includes clarifying key definitions/concepts and identifying key characteristics or factors relating to a concept. We excluded articles for one or more of the following reasons: literature reviews (not systematic or integrative) that did not contribute new concepts to the literature, did not study/discuss climate change, did not study/discuss one or more vulnerable populations, not related to specific mental health impacts, wrong publication type, not in English, and additional duplicates not detected by Covidence.

We developed and validated a data extraction chart built in Microsoft Word. Authors performed the data extraction independently and then results were compared and discussed to limit bias in the extraction stage. The resulting extraction table can be viewed in Appendix 1. The following data were extracted from the included articles: Bibliographic data (author, date, title, publication); Country of origin; Discipline; Aim/Purpose; Population and sample size/setting (if applicable); Methodology/methods/intervention/program; Outcome and how it was measured (if applicable); Key findings; Implications for practice (if applicable).

Following data extraction, we thematically coded the synthesis table. We then discussed the coding results and identified five themes for discussion. As an integrative review, no institutional review board ethical clearance was required.

We updated the search in July 2022. The same databases were searched with exception of Scopus since access to this database was discontinued for our team. The same procedures for screening, reviewing, and data extraction were used for the second analysis of manuscripts yielded.

## 3. Results

**Figure 2:**
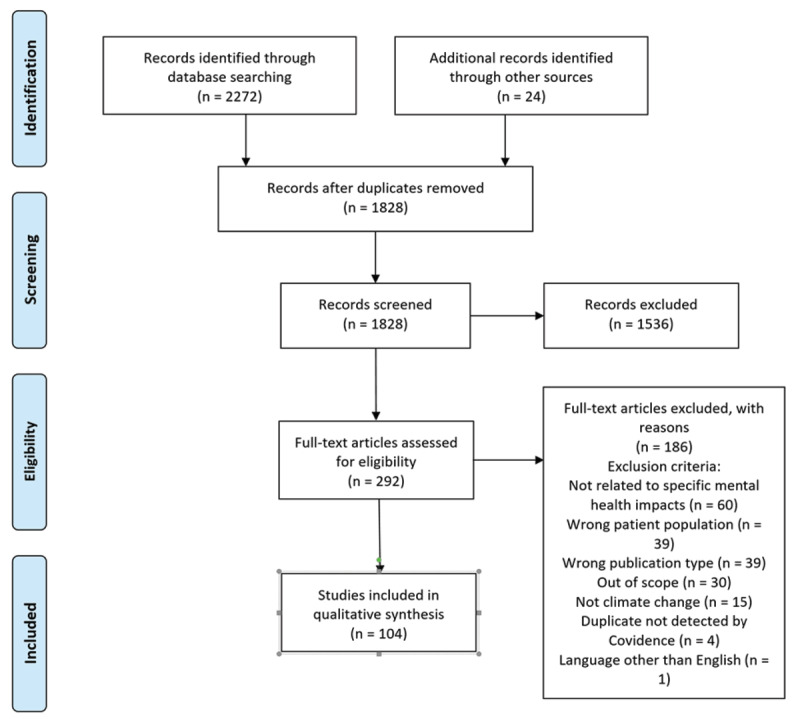
Study Flow Diagram.

The initial search yielded 2,272 results, of which 444 were duplicates that we removed. An additional 24 articles were identified for review through handsearching and through ancestry searches of reference lists. We reviewed the full text of 292 articles. After two rounds of screening, 104 articles remained for inclusion in the review. The updated search (July 2022) yielded 818 new records related to mental health and climate change. Covidence removed 52 duplicates. Thus, we screened 766 more titles and abstracts, assessed the full text of 199 more studies for eligibility, excluded 143, and ultimately included an additional 568 articles in this review. This was anticipated as a finding as there was a significant increase in the number of manuscripts published on mental health impacts of climate change beginning in 2020. The study flow diagram (Figure 2) is an illustration of the screening and inclusion process.

Qualitative methodology was among the most prevalent research methodology observed in the results (n = 21). Fewer articles reported mixed methods procedures (n = 14). And quantitative research was conducted (n = 23); however, the use of validated measures was inconsistent across studies. Theoretical framework articles, literature reviews, and policy/expert analyses were also retrieved when they offered a novel concept of the literature. Further results of this integrative review are summarized below and represent a synthesis of the full findings detailed in the Evidence Summary (Table 1).

The documented mental health effects of climate change among vulnerable populations globally in the retrieved results were numerous. *Depression* and *suicide* were frequently observed in the articles retrieved. For example, patterns of climate change have exacerbated farmers’ perceived risk of depression and suicide [26], marginalized groups had higher levels of suicidal ideation six months after severe flooding [51]. Additionally, results show increased prevalence of suicidal ideation and suicide plans [39]. Of note, people impacted by climate change who experience Major Depressive Disorder (MDD) may not benefit from normal course of treatment for this diagnosis [52]. ‘*Eco-anxiety*’ and *post-traumatic stress disorder (PTSD)* are also well-documented in the results. PTSD has been shown to be a direct effect of acute weather events [40], and traumatic exposure to major storms is predictor for multiple mental health impacts including substance use and dependence [53], particularly among people who already belong to marginalized groups [51]. Anxiety (and anxiety-mood) disorders both at baseline and in the follow up surveys among New Orleans residents following Hurricane Katrina were considerably higher than those found in previous surveys of mental illness after natural disasters in the US [39].

The previously novel mental health concept, *solastalgia*, has been presented by multiple studies to describe the myriad mental health effects related to climate change [9,32,40,50,54,55,56,96,131]. Nearly all articles retrieved described various forms of climate-related psychological distress experienced by vulnerable populations. These various forms of distress have been described as having three classes of psychological impacts: Direct (e.g., acute or traumatic effects of extreme weather events and a changed environment); indirect (e.g., threats to emotional well-being based on observation of impacts and concern or uncertainty about future risks); and psychosocial (e.g., chronic social and community effects of heat, drought, migrations, and climate-related conflicts, and post-disaster adjustment [25]. Psychological distress was noted across multiple populations – among adolescents [57,124,126], adults [31,36,58,122], older adults [38], people who have experienced climate migration [56,82,131], and among land-vulnerable persons [33,54,55,59,115,123]. Symptoms of psychological distress were described in the results as feelings of loss and grief, despondency, suffering despair, helplessness, hopelessness [43,48,55,135] and in sleep disturbances [59]. While not always labeled as such in the literature, the constellation of climate-related psychological impacts may nonetheless provide support that the prevalence of *solastalgia* is high across several vulnerable populations.

Across the lifespan, results showed that perhaps no groups are as vulnerable as children to the mental health impacts of climate change [23,30,37,44,45,47,57,60,61,62,80,83,84,86,87,91,96,97,100,101,104,106,108,109,114,117,118,121,127,132,133]. The youngest among us in society experienced several effects such as reduced capacity for learning, mental development (including from parasitic illness) following climate-related natural disasters [47,107]; greater negative affect and lower emotion regulation [37,102,103,128]; detrimental effects on infant temperament and early childhood [62]; and more overall restlessness and distraction [61]. Increases in anxiety behaviors (bed wetting, nightmares, clinging, aggressiveness) among children in low-income countries affected by climate changes were also observed [47]. Boys, indigenous children, and those from low-income households had worse direct mental health impacts, whereas girls showed more indirect mental health impacts (e.g., decreased ability to participate in organized physical activities) [61]. Adolescents acknowledged the mostly harmful mental health effects of events such as drought, but also demonstrated increased community connectedness as a result of drought [23], suggesting that resiliency and coping may be enhanced during climate stress. Pertinent to the wellbeing of children, results also suggest that parenting quality during climate crises may be altered, such as in increased parental stress response being noted towards children exhibiting anxious behaviors [47] and climate-related maternal depression and negative changes in the dynamics of mother-child dyads and families [62,116,117,118,120,124].

In addition to children, older adults are especially vulnerable to the mental health impacts of climate change [24,28,49,60,81]. Older adults are more likely to experience increases in cognitive difficulty during heat waves [59], and this was shown to cause feelings of “overwhelming, panic, anxious, concern, worried, desperate, fear” related to anxiety about extreme heat [28]. Extensive media coverage, such as on television news, add to anxiety levels among older adults [28].

While affected populations observed in results were diverse (such as reindeer herders in Sweden [27]), some similar groups were represented in multiple articles retrieved. First, people who are “land-vulnerable” either through occupation (farming, agriculture) or geographic location (drought-prone regions, low-lying coastal areas, frequent natural disasters) were highlighted in multiple ways [26,28,46,54,85,88,89,90,92,93,94,95]. Residents of rural Australia [34,63,64,98,119], vulnerable low-lying villages in East Malaita, Solomon Islands [31], and residents of New South Wales [29,64,99] provide just a few of the examples of geographic vulnerability represented in our findings.

Indigenous peoples, and the unique mental health effects faced by indigenous communities, were represented in multiple articles retrieved. Results revealed that indigenous peoples around the world contribute least to changes in the environment, yet they are disproportionately impacted for three reasons: location to vulnerable ecosystems (exposure); cultural and traditional lifestyles reliant on natural resources (sensitivity); and disproportionate economic distress [42]. Additionally, indigenous persons often experience the most significant health disparities (limiting adaptive capacity) [42,105,111,112,113]. This was true among indigenous persons globally, from Canadian Inuit persons [30,35], aboriginal and Torres Strait Islander Australians [50], and indigenous persons in Louisiana, US [32]. Though mentioned in only select articles retrieved, results also suggest that there are significant mental health effects related to climate-related relocation and migration [53,56,65,66]. Climate migrants have poorer mental health related to losing access to natural landscape and reduced sense of belonging [56,65], and also related to actual displacement and migration events [53,110,131].

## 4. Discussion

### 4.1 Varied Mental Health Responses

Results from this review demonstrate that the mental health responses to climate stress among vulnerable populations are varied, long-lasting, and affect many domains of life. Additionally, it is clear from results that the mental health effects of climate change events are less well understood than physical health effects. This appears to be related to several factors. Mental health outcomes are both direct and indirect [40]; they differ by type and severity of events; and are deeply contextualized by the social, cultural, historical settings in which they occur [52].

Retrieved articles show increased risk of specific mental health diagnoses related to climate change (e.g., depression or anxiety), but also suggest a pervasive or insidious constellation of mental health symptoms that some authors have identified as solastalgia. Among vulnerable populations, climate change often results in alienation, lack of belonging, and demoralization with or without specific psychiatric disturbances [52]. Psychological harm was also inflicted from damage to homes and possessions, global migration, added grief of losing/leaving loved ones, seeing parents and caregivers undergo stressful relocations, and breakdowns in social networks and economic security all appear to exacerbate feelings of solastalgia [9,40,45,50,96,131].

These life-altering events sometimes result in strong emotional responses leading to four maladaptive pathways: increase in drug and alcohol usage, increased family stress, increase in suicidal ideation, magnification of trauma [36]. As such, it is critical that healthcare providers work to mitigate the short, intermediate and long-term effects of climate change in relation to the mental health. Additionally, the grief process related to climate events may be altered, lengthened, or exacerbated by each client’s specific contextual stressors; a non-linear, dynamic approach to mental health treatment based on the client’s unique circumstance is indicated. Moreover, disaster recovery programs should be expanded beyond the immediate aftermath of significant events to include pre-disaster prevention strategies. It is especially important to consider this for vulnerable populations and the associated long-term mental health impacts on these populations [51,62,114]. The Substance Abuse and Mental Health Services Administration (SAMHSA) explored the concept of trauma and suggested that trauma results from an event, series of events, or a set of circumstances that is experienced by an individual as physically or emotionally harmful or life threatening and that has lasting adverse effects on the individual’s functioning and mental, physical, social, emotions, or spiritual well-being [67].

Inherent in the trauma-informed approach are four concepts: *realization, recognition, response*, and *resisting re-traumatization*. The first concept, realization encompasses a broad engagement at all levels of any organization and understanding of effects on patients, families, and communities. Second is recognition of signs of trauma by all health care providers. Third is the response to trauma experiences and building policy and advocacy approaches. Fourth is resisting re-traumatization of individuals, families, and communities. Key principles are important for mental health professionals which include safety, trustworthiness and transparency; peer support; collaboration and mutuality; empowerment, voice, and choice; and cultural, historical, and gender issues [67]. For optimal mental health care, integrating a trauma-informed approach in the setting of climate migration is key.

Climate change-related migration is an emerging and urgent problem as global locations are increasingly affected by wildfires, hurricanes with post-disaster relocation, high ambient heat temperatures with loss of arable land and agriculture as well heat stress, and sea level rise with water intrusion requiring migration [131,134]. Brown [68] wrote on behalf of the International Organization for Migration (IOM) and noted that there are two categories of climate-related migration: one involves *climate processes* and a second related to *climate events*.

#### The most vulnerable among us continue to be the most vulnerable to climate-related mental health distress

Among the themes that we identified as critical to mental health is that those most vulnerable to climate change are most likely to experience climate change-related mental health distress. A recent policy brief by the American Academy of Nursing on *Climate Change and Mental Health/Wellbeing* was published after completion of our integrative review which addressed the key clinical and health policy efforts that should be undertaken to address the most vulnerable, including children, and the elderly, and low-income populations who may have limited resilience [69]. From a clinical perspective, it is imperative to address mental health impacts of the most vulnerable which include anxiety, depression, post-traumatic stress disorder, and suicide and suicide ideation. Carleton [70] notes that heat stress due to high ambient heat temperature may be responsible for 60,000 additional suicides in India. It is important to note that farmers have high rates of anxiety, emotional distress, depression, and suicide risk [69].

It is well established that climate change disproportionately affects the most vulnerable in the US and globally and that hurricanes and climate-associated disasters, air quality issues, and other climate-related health consequences most affect Black and Brown communities [71,72,73,133]. Recent literature related to poor air quality indicates that women experience poorer pregnancy outcomes including preterm labor, low-birth weight, and stillbirth due to exposure to unhealthy air and high ambient temperatures [74] and that Black women are disproportionately affected [75]. It is also important to note that health consequences in our climate-changing world are a major issue in global climate-related conflict and migration and associated with mental health sequelae [5].

### 4.2 Threats to Livelihood Are a Predictor of Mental Health Distress

Populations who are land-vulnerable (dependent upon land and natural resources for livelihood) are particularly susceptible to the effects of climate change. The impacts and threats to livelihood lead to significant mental health consequences including anxiety, depression, hopelessness and suicide [26,28,46,55]. Farmers and farm workers in rural areas were the most well-represented land-vulnerable population in the literature [26,28,34,46,54,59,115,123]. The most impactful and well-documented manifestation of climate change was drought and lack of water resources [24,25,54,55,59,64,115,123]. Finally, the challenges that climate change presents to the livelihood of those dependent on land impacts identity and creates a significant loss of connection to land and sense of place [26,27,54,64,125,129,130]. Drought was the most well-documented consequence of climate change. Drought leads to significant impacts on populations who depend on land for livelihood, with detrimental consequences for mental health. Populations whose livelihood depends on natural resources also feel a strong sense of identity and connection with the land and a sense of place. It is important to understand how a loss of sense of place secondary to climate change may impact mental health.

Health care providers must consider patients’ mental health in the context of their life circumstances. Acting as advocates for our patient’s mental well-being, it is important to be familiar with screening tools and government resources to assist with whatever challenges they may be facing. Recognizing patients who are land-vulnerable in the setting of an ongoing or sudden climate event, clinicians can be prepared to help with new mental health concerns or manage exacerbations of chronic mental health issues that may be triggered by the additional stress of consequences of climate change. Mental health screening tools such as Environmental Distress Measure, and implementation of programs such as Caring for Country projects may be helpful in acknowledging trauma and implementing community intervention [32,41,64].

#### Sociocultural influences impact mental health and well-being within the context of climate change

Most that discussed the impact of climate change on social connectedness focused specifically on Indigenous Peoples and the ways in which climate change can disrupt their relationship to both natural environment and culture and worsen existing mental health disparities [30,32,35,41,50,64,76,129,130]. Within Indigenous communities, there is an inter-relationship between and among environment and culture and mental wellness and social cohesion [30,35,129]. The Inuit, for example, have an eco-concept of self and see the environment as underlying all social determinants of health and wellbeing [35]. Moreover, social relationships are an important part of Inuit culture, and social connections are critical for mental wellness. Land is essential to social support networks; therefore, climate change impacts that threaten land, and in turn, social connections, are deleterious for individual mental health and wellbeing [35]. In fact, exposure to environmental change and decreased social support predicted poor mental health outcomes across the populations identified in these studies including Indigenous peoples, older adults, and climate migrants [32,56,77]. Moreover, discrimination and marginalization of Indigenous peoples lead to poor mental health outcomes [32,50]. Hunter [50] discusses how marginalization by non-Indigenous individuals can lead to feelings of self-blame, low self-esteem as well as increased violence, and self- harm.

### 4.3 Screening/Health Interventions for Vulnerable Groups Are Critical

Screening and health interventions for vulnerable groups before and after severe climate events is critical, particularly for psychological and mental health distress. Emerging terms in the literature related to anticipatory mental health issues from the effects of climate change are *solastalgia* and *eco-anxiety*. Due to increased risk of mental health problems following climate-related events, screening for solastalgia with at-risk and indigenous populations is recommended [42].

Prior to climate events, communities can engage in routine education and screening strategies to promote awareness and mitigate effects of air pollution in homes and communities [60,135]. Prevention-focused strategies and interventions should consider culturally relevant and integrative mental healthcare delivery models [76]. Strategies may include public health agencies creating both universal and targeted interventions for communities, the development of health equity councils, inclusion of local minority health directors and partners in key decisions and appreciating the disproportionally high rates of pre-existing mental illness in vulnerable populations exacerbated by climate change [65].

Mental health awareness should be addressed both pre-disaster and long-term following a severe weather event. Interventions aimed at addressing and promoting resilience of vulnerable populations with underlying mental health conditions as well as early assessment and response proximal to the climate event may be effective in preventing the onset of symptoms and promoting recovery [51,65,129,132]. Examples of pre-disaster interventions include mental health screening for pregnant individuals as well as screening for indigenous and other vulnerable populations at risk for natural disasters and with existing health inequities [32,62,125], disaster planning at the community level [53], and policy development appreciating the potential mental health impacts of extreme heat on vulnerable individuals [61]. While many disaster relief programs are aimed at immediate intervention, these efforts must be extended due to the persistent mental health problems following the immediate aftermath [51,132,135].

Literature reviewed acknowledged the direct and indirect impact of climate change on development and exacerbation particularly of depression and suicide, anxiety, substance use disorder, post-traumatic stress disorder, and solastalgia [6,10,34,36,40,42,62,70,131], It is also important to note that in our previous work addressing mental health sequelae of climate change [78,79], there is an increasing body of knowledge about the importance of interprofessional approaches to address mental health sequelae of climate change—particularly in the ED; and that developing frameworks have broad application for use by all health professionals and are important for relevance in clinical practice.

## 5. Conclusion

This integrative review yielded 104 relevant articles that provided insight into the mental health impacts of climate change among vulnerable populations globally. Results suggested that land-vulnerable persons, Indigenous persons, children, older adults, and climate migrants are disproportionately affected by climate change-related mental health sequelae, including solastalgia, suicidality, depression, anxiety/eco-anxiety, PTSD, insomnia, substance use, and behavioral disturbance. The VPCM provides a structure for providers to understand the mental health needs of patients before, during, and after climate events (Table 2). This model has unique relevance for health care professionals and community leaders in addressing the myriad of mental health sequelae related to climate change.

**Table 2:** The Vulnerable Populations Conceptual Model (VPCM) With Application to Climate Change and Mental Health (Flaskerud & Winslow, 1998).


RESOURCES AVAILABLE	RELATIVE RISK	HEALTH STATUS

Infrastructure, clean water, clean air, stable temperature, access to energy, sanitation, hygiene, access to safe food and nutrition, transportation Economic support during extreme climate events and/or for populations affected by climate-related income loss Community engagement and social support, including the role of elders and community leaders Local, national, and international public- and private-sector relief organizations Organizations providing support and services to Indigenous persons, including established Indigenous Nations School nurses, counselors, teachers, and leaders	Health impacts of climate change: injury, death, mental health impacts, population displacement, waterborne diseases, higher economic costs of recovery, infrastructure damage, ecosystem changes, asthma, cancer, CV, heat effects, human development effects, neurological, vector-borne and zoonotic diseases (NIEHS, 2019) Solastalgia is common, and represents the myriad symptoms of climate-related psychological distress present throughout the literature Other mental health impacts include increased suicidality, depression, anxiety/eco-anxiety, PTSD, insomnia, substance use, and behavioral disturbances Median years of potential life lost in people with mental disorders: 10 years (Walker, McGee & Druss, 2015) Individuals in extreme weather have higher rates of morbidity (illness) and mortality (death) (NIEHS, 2019)	Provider screening for suicide, depression, anxiety, substance use, and PTSD; follow-up mental health care when present Screening for the myriad symptoms that indicate solastalgia; follow-up mental health care when present Preventative care before climate events is ideal (e.g., in areas prone to routine climate events) Mental health care should be extended beyond brief post-disaster interventions, as effects of adverse events are long-lasting and insidious Psychological First Aid (before and after specific climate events) for vulnerable populations is indicated Consider a trauma-informed approach to care Validated measures should be used for symptom assessment when possible, such: Universal suicide risk screening; PHQ-2: Depression; GAD-2: Anxiety; NIDA: Substance use; Subjective Units of Distress Scale: Intensity of distress; PC-PTSD-5: PTSD (Nicholas et al., 2020)


## Additional File

The additional file for this article can be found as follows:

10.5334/aogh.4105.s1 Appendix 1. Results Table (n = 104) Reference number in brackets.
