# Supplementary material for: Mental Health Impacts of Climate Change Among Vulnerable Populations Globally: An Integrative Review
Source: Ann Glob Health. 2023 Oct 6;89(1):66. doi: 10.5334/aogh.4105 (PMC10558031; doi:10.5334/aogh.4105)
Supplement: Appendix 1. — Results Table (n = 104) Reference number in brackets. [file agh-89-1-4105-s1.pdf]

Appendix 1. Results Table (n=104)
Reference number in brackets

Author, Date	Aim/Purpose	Relevant Population	Methodology / Methods / Intervention / Program	Key Findings Related to Review Question	Implications for Practice for Health Professionals
Alston (2012)<br><br>[46]	Examine research conducted over years with rural Australian men working on farms and suicide	Adults: Rural Australian men	Expert analysis	The rate of suicide among Australia's rural men is significantly higher than rural women, urban men or urban women in climate-changing world.	Providers should assess for indicators of suicidality, including higher levels of social isolation, vulnerable socio-economic circumstances, and ready access to firearms.<br><br>Another factor is the challenge of climate transformation for farmers.<br><br>Health and well-being of rural men requires an understanding of the cultural context, gender relations/roles, and a dominant form of masculine hegemony that lauds stoicism in the face of adversity
Anderko & Pennea (2022)<br><br>[80]	Review the health impacts of climate change on children and describe the role of nurse practitioners in addressing these impacts.	Children	Discussion paper	Mental health issues in children including depression and post-traumatic stress disorder are increasing as a result of climate change-related natural disasters. Hopelessness and pessimism are also seen in many young people; and the intensity and frequency of extreme weather events may exacerbate mental health disparities for those living in low-income, marginalized communities.	Providers should consider following recommendations from the National Association of Pediatric Nurse Practitioners, which include: integrating anticipatory guidance, prevention strategies, standardized screening, and early identification of mental and behavioral health issues into routine primary pediatric care.
Asugeni et al. (2015)<br><br>[31]	Examine mental health issues related to sea-level rise for people in East Malaita, Solomon Islands	Sixty residents of six vulnerable low-lying villages in East Malaita, Solomon Islands. Solomon Islands has one of the highest rates of sea-level rise globally	Quantitative and qualitative questionnaires were administered to 10 residents of each of the six vulnerable villages	Four themes emerged: 1) experience of physical impacts; 2) worry about the future; 3) adapting to sea-level rise; and 4) government action.<br><br>Primary mental health symptoms reported included anxiety and insomnia<br><br>Of 57 participants, 56 stated that the worry about sea-level rise affected their thinking and behavior and that of their families and communities. More than half identified negative effects on themselves and their communities.	Mental health providers should be informed about the needs of people in vulnerable geographic regions<br><br>Policy and health service responses should be provided at the local level<br><br>Providers should assess for anxiety and insomnia

Ayalon et al. (2021)<br><br>[81]	Describe three themes that climate change creates inequities among older adults	Older adults	Invited perspective paper	First, climate change increases vulnerability of those with current mental health conditions—are at increased risk for limited social support during natural disasters which limits social connection.<br>Second, climate change triggers new mental health conditions. Climate change disproportionately affects the mental health of older persons resulting in shock, PTSD, phobic impairment, SUD, suicidality, aggression<br>Third, climate change ageism threatens well-being and can lead to feelings of guilt, powerlessness.	The authors call for a United Nations convention on the rights of older adults to live free from discrimination and have full enjoyment of their human rights, integrating environmental sustainability
Ayeb-Karlsson (2020)<br><br>[82]	Explicate understanding of the concept of Trapped Populations by examining climate-induced socio-psychological immobility and its link to internally displaced persons wellbeing	IDP in a slum of Dhaka Bangledesh (the Bhola Slum) who were forced to leave Bhola Island and who are trapped in an environmentally high-risk area because of economic constraints.	Q-methodology and Discourse analysis over 3 years. N=62 participants. Snowball sampling, but authors attempted to fully represent characteristics of group. Q-statements to capture subjective attitudes were developed based on previous qualitative research on urban immobility wellbeing	Five discourse groups were identified:<br>The landless: I want to return, but the erosion took my land—leading to negative impacts on their lives and future<br>The Displaced: This is not where I belong. I want to go home.<br>The sacrificed: Lost health and honour for economic gain.<br>The returners: I came here to save up money, after that I will return home.<br>The dreamers: urban dreams of betterment<br>Non-economic losses across the 5 discourses included loss of identity, honour, sense of belonging, physical and mental health or wellbeing.	When mobility is framed as an adaptive policy solution the following questions need to be asked; whom is the solution for and by whom is it raised? Why is there an over-representation of rural populations who are trapped? Why is climate induced migration/mobility more commonly studied than climate-induced immobility?<br><br>Political and financial efforts must be undertaken to ensure climate-induced migrants and immobile populations have immediate access to psychological support to help heal trauma related to migration and adjust to their new living conditions.
Barkin et al. (2021)<br><br>[83]	Synthesize the literature related to childhood survivors of extreme weather events to describe psychological symptoms and behavior, potential for long-term effects and potential protective and risk factors.	Children	Commentary	Post traumatic stress is the most common mental health consequence in child survivors of extreme weather events and is often co-morbid with depression and/or anxiety, particularly existential anxiety.<br>Others include aggression, attachment insecurity, diminished academic performance.<br>Potential long-term effects: persistent symptoms of trauma, anxiety, grief, loss increasing cumulative stress<br>Protective factors include coping efficacy and acculturation, family resilience and social support, family functioning and post-traumatic growth, and positive	Despite high reports of post-traumatic symptoms, a small number access counseling.<br><br>Techniques including dispositional mindfulness, deliberate rumination, and positive reappraisal should be investigated further.

				reappraisal, household air conditioning and high levels of neighborhood greenspace.<br>Risk factors: intrusive rumination, disaster-related loss, absence of parent/caregiver	
Barkin et al. (2022)<br><br>[84]	Explore the importance of mental health in the perinatal period with emphasis on climate change-related stressors	Perinatal women	Commentary	There is a link between extreme weather event exposures, perinatal health, and mental health. Trauma may occur from potential displacement and interruptions in care.	Certified nurse midwives, advanced practice RNs, Psychiatric Mental Health NPs, and OB/GYN and public health nurses must be aware of the connection between climate change and mental health and institute screening protocols for anxiety, depression, and SDoH
Bartlett (2008)<br><br>[47]	Extrapolates existing knowledge in a number of related areas to present a picture of the probable implications for children's health, safety, and long-term well-being, especially in lower-income countries and communities that are at highest risk from climate change.	Children	Expert analysis	After climate disasters, children exhibit anxiety behaviors (bed wetting, nightmares, clinging, aggressiveness) and this may contribute to abusive response from stressed caregivers<br><br>Climate change could erode capacity for learning, mental development in children.	Providers should recognize children's mental health vulnerability to climate change and climate disaster both directly (e.g. anxiety) and indirectly (e.g. abuse and neglect from stressed caregivers)
Batterham (2022)<br><br>[85]	Synthesize quantitative evidence for the relationship between environmental factors and mental health and well-being in rural areas	Adults living in rural areas	Systematic review	N=28 studies. Most in Australia, with some in Europe, USA, Asia—all within the past 15 years. High levels of mental ill health among people living in rural areas was a general finding. There was typically a relationship with extreme climate or weather or environmental degradation. Poor outcomes when area related such as drought; however, this was not found at the individual level suggesting differences in response to the event.	Gaps exist in the literature remain on how rural environments influence mental health and well being.
Benoit (2022)<br><br>[86]	Analyze lay press narratives about how youth respond to climate change to examine dynamic	Children and teens	Scoping review: qualitative analysis of lay literature	Highlights ecological grief, ecoanxiety and how youth may leverage for environmental action. Adult response to youth mental health re: climate change is rarely addressed in the lay literature.	Mental health providers caring for children and teens should be aware of lay media's narrative re: youth's reactions to climate change and environmental degradation. Existential psychology can help individuals

	between minors and adults related to climate crisis			Themes identified in children: fierce young activists; adultified children; innocent victims, and/or ultimate saviors. Themes identified in parents/adults: Experiencing ecoanxiety through adulthood; managing children's ecoanxiety; criticizing youth-led activism; and transcendence: reimaging climate actin, our word, and ourselves	confront difficult truths. Youth can be empowered by taking action—parents and adults can encourage this.
Berry, Bowen, & Kjellstrom (2010)<br><br>[40]	Examine the importance of the possible impacts of climate change on mental health and to propose a framework for better understanding on how climate change may affect mental health	Adults	Framework Development/ Proposal	Developed framework showing putative causal pathways linking climate change and mental health with findings that are direct and indirect effects of climate change<br><br>Climate change related disasters affect communities, physical health, and mental health<br><br>High risk of PTSD related to climate change related acute weather events<br><br>Subacute weather events (heat/drought) also have impacts on depression and trauma.	Providers should assess for PTSD, depression, and solastalgia.
Berry et al. (2010)	Describes how 'caring-for-country' (civic engagement) projects may provide a novel way to achieve the linked goals of climate change adaptation and co-benefits for remote Aboriginal social and emotional wellbeing"	Adults in remote Aboriginal Australian communities	Framework Development/ Proposal	Conceptual model theorizing that caring for country would strengthen social capital which would in turn be associated with improved social and emotional wellbeing, creating positive feedback loops where better health would contribute to enhanced social capital and greater engagement with caring for country	Caring for country projects can better mitigate the mental health impacts of climate change
Billiot (2017) [32]	Explores relationship between environmental changes and indigenous health outcomes in Louisiana after	Adults in 57 United Houma Nation in south Louisiana	Mixed methods	Significant relationship between exposure to environmental changes and poor mental health.<br>Environmental change exposure significantly predicted meeting criteria for a mental health diagnosis.<br>Discrimination, exposure to	Mental health screenings and environmental assessment are indicated in Indigenous persons

	controlling for moderating effects of indigenous-specific factors of historical trauma, discrimination, and social support			environmental change and decreased social support predicted poor mental health .	
Billiot & Mitchell (2019)<br><br>[42]	Describe a model examining the impact of environmental change on indigenous health and well being	Indigenous populations	Framework Development/ Proposal	Indigenous peoples around the world contribute least to changes in the environment yet they are disproportionately impacted due to: 1) location relative to vulnerable ecosystems (exposure); 2) cultural and traditional lifestyles reliant on natural resources (sensitivity); and 3) an existing vulnerability (among the poorest of their societies) have greatest health disparities (adaptive capacity)	Professions focused on improving the overall health and well-being of society should be concerned with the general well- being of the earth<br><br>Theories used in multiple disciplines can merge to advance our understanding of the long-term impacts<br><br>Transdisciplinary work can pave the way for improved outcomes for indigenous persons experiencing climate change
Buthmann et al. (2019)<br><br>[37]	Examines the association between disaster-related pre- and post-natal maternal stress and offspring temperament at six months old	380 pregnant women who experienced Superstorm Sandy in 2012	Quantitative/ prospective cohort before, during, or after Superstorm Sandy	Length of time without phone or electricity and financial loss was associated with offspring negative affect<br><br>Financial loss and threat of death or injury was associated with emotion dysregulation<br><br>Offspring born before the storm had greater negative affect and lower emotion regulation than those born after the storm	There is a need for more data on pre-existing and post-disaster stress in mothers and fathers.
Carnie et al. (2011)<br><br>[23]	Explore the drought-related experiences of young people and to contrast these with their teachers' and other adults' observations	Young people, their teachers and service providers in rural schools in NSW Australia	Youth and community forum data were qualitatively analyzed (organized under the Rural Adversity Mental Health Program)	Participants welcomed increased community connectedness in response to prolonged drought but reported that drought's mental health impact was mainly negative<br><br>Adults observed children's distress (worrying about their families, increasingly isolated, at risk of harm, unable to obtain help and facing educational and employment limitations) and wondered whether others had observed it	Providers and school professionals should coordinate support within schools, schools working together, more dialogue, about mental health and where to seek help for them and their friends, and access to support people who understood drought and rural circumstances and on whose discretion they could rely<br><br>Mental health programs that are developed in and for metropolitan contexts need to be adapted before being deployed in rural settings

				Young people disclosed mental health and relationship difficulties at school and at home; worry about their families, communities and futures; about money; and being isolated	
Chan et al. (2018)<br><br>[38]	Examine short-term associations between temperatures and mental disorder hospitalizations in a subtropical city with a mean annual temperature over 21 ◦C	Adults in Hong Kong seeking mental health care	Quantitative	Positive temperature–mental-disorder admissions were significantly associated in a warm subtropical region and the association was most prominent among older people. Daily mental disorder hospitalizations between 2002 and 2011 in Hong Kong (n= 44,600) were regressed on daily mean temperature, relative humidity, and air pollutants, adjusted for seasonal trend, long-term trend, day-of-week, and holiday. Analyses were stratified by disease class, gender and age-group	Targeted strategies for persons in subtropical cities who are at risk for mental health hospitalizations, particularly older adults
Clemens (2022)<br><br>[87]	Explore the mental health impacts of climate change within the Intergovernmental Panel on Climate Change report	Children and Adolescents: In Europe	Expert analysis	Mental health can be impaired by a variety of types of consequences: direct consequences such as disasters; indirect consequences, such as loss of land, flight and migration, exposure to violence, change of social, ecological, economic or cultural environment. Awareness of the existential dimension of climate change in children and adolescents can influence their well-being or challenge their mental health.	Consequences of climate change for somatic health may interact with mental health or have psychological sequelae in children and adolescents.
Cooper et al. (2019)<br><br>[24]	Addresses the wellbeing of a marginalized population living with continuing environmental change to explore a spectrum of perspectives on water security and wellbeing	Adult participants in Afar Region, Ethiopia	Qualitative	Vulnerable individuals who depend on land/natural resources for livelihood are particularly vulnerable to the effects of climate change, including the mental health and psychological distress associated with lack of water resources	Providers should recogniz(e) the influence climate change has on mental health among vulnerable populations not only from a wellbeing and equity perspective, but also in terms of how it shapes a population's ability to cope and adapt to environmental change
Cunsolo Willox et al. (2012)	Explores the feelings and experiences related to climate	Adult Nunatsiavut Indigenous peoples	Mixed methods	Climate change negatively affects feelings of place attachment by disrupting hunting, fishing, foraging,	Further study needed in indigenous communities

[33]	change among people in rural hunting/fishing regions			trapping, and traveling, and changing local landscapes. These changes subsequently impact physical, mental, and emotional health and well-being.	More context-specific climate-health planning and adaptation programs are needed<br><br>Providers should understand place-attachment as a vital indicator of health and well-being and recognize climate change as an important determinant of health
Dean & Stain (2010)<br><br>[57]	Authors sampled and re-sampled adolescents from the same rural area and determine if this resilience remained after ongoing drought three years later	Adolescents male and female of New South Wales	Mixed methods	Adolescents reported significantly higher levels of emotional distress than those in the previous study.<br>Thematic analysis showed consistency with the previous study as well as new themes of grief, loss and the impacts of global climate change	A preventative intervention with a focus on family and community is recommended to address the mental health of adolescents enduring a chronic environmental adversity such as drought
DiGiorgi (2020)<br><br>[88]	Examine the experiences of sample of migrants in Italy from African countries with two groups: one from countries with extreme vulnerability to climate change,, and the second from countries with high vulnerability.	Adults: Migrants in Italy	Quantitative	Migrants from countries with extreme exposure to climate change perceived the greater vulnerability of their home country and associated with less availability of water and electric power. Both groups (extreme vulnerability and high vulnerability) indicated that there were aspects of climate change that are most strongly felt related to dependence on food supply and the lack or decreased availability of electricity.	Findings suggest that social capital is important for those impacted by extreme and high vulnerability to climate change. Loss of social capital and emotional disorders were an important finding, There was a significant but weaker correlation between perception and emotional disorders. Thus social capital was found to be a useful intermediate variable between climate change and mental health. Efforts aimed at preserving social capital can mitigate the impact of climate change on mental health.
Doherty &Clayton (2011)<br><br>[25]	Differentiate between types of psychological impacts of climate change	Adults	Expert analysis	Three classes of psychological impacts of climate change: direct (e.g., acute or traumatic effects of extreme weather events and a changed environment); indirect (e.g., threats to emotional well-being based on observation of impacts and concern or uncertainty about future risks); and psychosocial (e.g., chronic social and community effects of heat, drought, migrations, and climate-related conflicts, and post-disaster adjustment)	Psychological interventions in the wake of acute impacts are indicated<br><br>Providers should work to reduce vulnerabilities contributing to severity of impact; promote emotional resiliency and empowerment in the context of indirect impacts; and act at systems and policy levels to address broad psychosocial impacts
Dumont (2020)<br><br>[89]	Explore climate change as linked wth adverse mental health effects, and elevated rates of suicides observed in some	Adults: refugees	Expert analysis	Studies demonstrate a population-wide increased risk of suicide of up to 3% within a few days after episodes of significantly worsened air quality. Aid pollution is a major contributing factor with increased risk of suicidality	It is critical that psychiatrists and mental health professionals become familiar with the climate mental health literature and develop plans of care to address climate mental health issues.

	communities affected by climate change. There are increasing rates of suicide in the US as climate change intensifies.			worsened with poorer air quality.	
Edwards et al. (2015)<br><br>[34]	Examine the mental health of rural Australia residents (farmers and non-farmers)	Adult residents of rural and regional Australia	Quantitative survey	Drought has negative mental health impacts, particularly among farmers and farm workers. The more severe the agricultural impact of drought, the greater the impact on mental health<br><br>Farmers who reported that the drought had eliminated or reduced their farm's productivity to the lowest point ever had significantly higher rates of mental health problems and lower mental health wellbeing scores than those who did not report they were in drought or reported that the drought had little or no effect	Providers should assess and provide care for rural farmers impacted by drought. Mental health impacts of drought require further study.
Ellis & Albrecht (2017)<br><br>[26]	Determine if and how 'sense of place' and mental health are affected by a changing climate	Adults in the Western Australian Wheatbelt	Qualitative	Community-based qualitative case study (three-part interview series with 22 family farmers and 15 interviews with various agricultural and mental health key informants). Patterns of climate change have exacerbated farmers' worries about the weather, undermined notions of self-identity, and contributed to cumulative and chronic forms of place-based distress, culminating in heightened perceived risk of depression and suicide	Suicide and depression screening are critical for drought-affected populations.<br><br>Providers should understand how 'sense of place' may impact clients' mental health related to climate change.
Enson (2019)<br><br>[44]	Reviewed literature related to the impact of climate change on young people	n/a	Review article	The climate crisis is a child rights crisis and is the greatest threat facing the next generation, threatening children's rights to protection and education<br><br>The environmental challenges and uncertainties facing children and young people can have a profound impact on their mental health and wellbeing	Providers should assess impact of climate change on young people, particularly related to personal safety, access to education, and mental health.
Evans (2019)<br><br>[48]	Addresses what is known about behavioral impacts of climate change and	n/a	Expert analysis	Behavioral impacts of global climate change include negative affect, interpersonal and intergroup conflict, and possibly psychological distress increase	There is a critical need for further examination of behavioral impacts of climate change. Barriers to further investigation include: lack of data on direct experience

	identifies barriers to further investigation			with rising temperature<br><br>Droughts, floods, and severe storms diminish quality of life, elevate stress, produce psychological distress, and may elevate interpersonal and intergroup conflict<br><br>Recreational opportunities are compromised by extreme weather, and children may suffer delayed cognitive development<br><br>Elevated pollutants concern citizens and may accentuate psychological distress<br><br>Outdoor recreational activity is curtailed by ambient pollutants	with climate change rather than indirect assessments related to projected changes; poor spatial resolution in environmental exposures and behavioral assessments; few rigorous quasi-experimental studies; overreliance on self-reports of behavioral outcomes; little consideration of moderator effects; and scant investigation of underlying psychosocial processes to explain projected behavioral impacts
Filiberto et al. (2008)<br><br>[49]	Addressed specific types of climate threats and the effect of climate change on older adults	n/a	Expert analysis	Extreme weather events, exacerbated vector-borne diseases, compromised agriculture, reduced availability of fresh water, and decreased habitability of human population centers are the likely effects of unabated climate change and older adults are most vulnerable<br><br>The evidence that climate change will potentially adversely affect older people disproportionally is compelling.	If the rapidly increasing older population worldwide is to be protected from the effects of climate change to the greatest degree possible, greater knowledge is our most important weapon<br><br>More research regarding the specific effects of climate change on older adults is needed
Ford (2020)<br><br>[90]	Explore Indigenous peoples globally related to significant exposure to climate change and are at-risk despite evidence of resilience.	Indigenous peoples	Perspective/discussion paper	Common factors affecting resilience of Indigenous peoples include the interconnected roles of place, agency, institutions, and collective action. Indigenous knowledge, and learning help	Indigenous peoples have adaptation and coping abilities to adapt to environmental change. Relationships with place are essential for Indigenous peoples to offer a foundation for belief systems, identity, knowledge, and livelihood practices..
Fortuna (2020)<br><br>[91]	Examine disasters and impacts on children which may lead to mental health including increased risk of a variety of mental health sequelae.	Children	Discussion paper	Psychological effects of climate change impact children and their resilience, vulnerability and have inequitable impacts.	Climate change results in disasters that have direct and indirect effects of climate change, including geographic displacement, with resulting mental health impacts for children that include PTSD, depression, anxiety, phobias, sleep disorders, attachment disorders, and substance abuse.

Fritze et al. (2008)<br><br>[43]	Examine the direct and indirect impacts of climate change such as extreme weather events	n/a	Expert Analysis	The direct impacts of climate change such as extreme weather events will have significant mental health implications<br><br>Climate change is already impacting on the social, economic and environmental determinants of mental health with the most severe consequences being felt by disadvantaged communities and populations	Providers should understand the full extent of the long term social and environmental challenges posed by climate change, and the potential they have to create emotional distress and anxiety<br><br>Providers should understand the psychosocial implications of climate change as an important starting point for informed action to prevent climate change impacts at the individual and community levels<br><br>The concepts of climate-related hope and despair require further study
Fuentes (2020)<br><br>[92]	Examine the impact of climate change and natural resource exploitation on Indigenous people's well-being	Indigenous communities in Eastern Canada	Quantitative	Indigenous peoples are impacted by climate change through reducing access to ecosystem services, which subsequently impacts transmission of traditional knowledge leading to mental health problems. Both mitigating effects of sociodemographic characteristics (i.e., age, gender, parenthood, and time spent on the land) and protective factors (i.e., health, quality of life, resilience, life on the land, life in the community, and support from family and friends) were considered in this study.	Results suggested that impacts of environmental climate change increased with age however were lower for participants with higher quality of life. Resilience outcomes were opposite expectations in that more resilient participants reported more mental health impacts. The authors suggest that this could be because less resilient individuals remained away from the land when environmental changes were excessive while the most resilient participants identified significant impacts of climate changes.
Fuller (2022)<br><br>[93]	Explores the politics of urban climate responsibility in Hong Kong and Singapore	n/a	Discussion paper	Cities have a responsibility to act on climate change, yet the approaches to this responsibility are complex, and the role of international and regional aspects are complex in addressing climate responsibility.	The Asia Pacific region is heavily impacted by climate change and this discussion paper addresses the geographies of urban climate responsibility with a focus on attribution, production and special impacts with implications for climate justice and resilience in the Asia Pacific region.
Furberg et al. (2011)<br><br>[27]	Explores the experiences and perceptions of climate change among Swedish reindeer herding Sami	Swedish reindeer-herding Sami persons	Qualitative interviews	One core theme emerged: facing the limit of resilience. Swedish reindeer-herding Sami perceive climate change as yet another stressor in their daily struggle<br><br>Herders have experienced severe and more rapidly shifting, unstable weather with associated changes in vegetation and alterations in the freeze thaw cycle, all of which affect reindeer herding. Forecasts	Reindeer herders are particularly vulnerable to climate change impacts, including serious consequences for trade and overall way of life

				about climate change from authorities and scientists have contributed to stress and anxiety<br><br>Some adaptive strategies are discordant with the traditional life of reindeer herding, and there is a fear among the Sami of being the last generation practicing traditional reindeer herding	
Galway et al. (2019)<br><br>[9]	Identifies the existing literature on solastagia	n/s	Literature review	Solastalgia is a new term and refers to a new concept for understanding the links between human and ecosystem health, and the expansive and cumulative impacts of climatic and environmental change on mental, emotional, and spiritual health	There is a need for additional research employing diverse methodologies, across a greater diversity of people and places, and conducted in collaboration with affected populations<br><br>There is a need for greater attention to the practical implications and applications of solastalgia research
Galway (2022)<br><br>[94]	Examine the views of community members in Fort Williams First Nation via interviews with the Two-Eyed Seeing approach centering on working collaboratively with Indigenous and non-Indigenous knowledge and peoples	Fort Williams First Nation peoples in Ontario	Qualitative	Five themes emerged in this qualitative survey of Indigenous elders: 1) observations and experiences of changes on the land, 2) lack of care and respect for Mother Earth as the root cause of climate change, 3) healthy land, healthy people, 4) youth and future generations, and 5) (re-)connecting with land and culture.	’All our Relations’ is a thread woven across these themes emphasizing that climate change is understood and experienced through relationships and relationality.
Gibbs et al. (2013)<br><br>[63]	Examine via longitudinal study regarding the medium to long term impacts of bushfires on mental health and social connectedness.	Communities affected by bushfire	A longitudinal mixed method	Conducting the study over five years and focusing on the role of social networks will provide new insights into the interplay between individual and community factors and their influence on recovery from natural disaster over time. The study findings expand understanding of long term disaster recovery needs for individuals and communities.	Longitudinal results suggest an impact of social networks in limiting mental health consequences.
Gibson (2020)<br><br>[95]	Examine if climate change affects individuals’ mental	Adults in Pacific Island atoll nation	Mixed methods	Participants’ experiences of distress related to types of stressors and demonstrated that a high proportion of	Climate change is a significant risk to mental health and requires decision-makers to consider these risks when examining climate-

	health in the small island developing state of Tuvalu, a Pacific Island nation exceptionally vulnerable to climate change.			participants are experiencing psychological distress that reportedly cause them impairment in one or more areas of daily life.	related harms and considering the costs of inaction.
Gislason (2021)<br><br>[96]	Explore impacts on children and youth showing increasing levels of mental health distress due to the climate crisis, with feelings of sadness, guilt, changes in sleep and appetite, difficulty concentrating, solastalgia, and disconnection from land.	Children and youth	Qualitative	Children and youth experience direct and indirect effects from climate change and this impacts their mental health. This population has varied perceptions of climate change based on their social locations with solastalgia and eco-anxiety as negative outcomes.	Mental health impacts of climate change are significant adverse outcomes on children/youth and are tied to Social Determinants of Health (SDoH). A second deleterious impact is related to the Ecological Determinants of Health (EDoH).
Godden (2021)<br><br>[97]	Examines the intersection of mental health, climate change, and the role of climate activism as a resilience approach, as well as the mental health impacts.	Children and youth in First Nation in western Australia	Discussion paper	The results focus on colonialism, capitalism and dangerously high emissions and the importance of strengthening the capacity of parents, adults and youth and mental health resources.	The intergenerational injustices of climate change suggest that children and young people are most at risk from mental health sequelae of climate change, however have the least power to limit climate change. Future generations of children and young people will continue to urge that climate justice be a key focus.
Guerrero et al. (2013)<br><br>[76]	Analyzes the biological, psychological/psychocultural, and social and environmental impacts (risk or protective) on either the prevalence/presentation of mental illness, help-seeking behavior or access to mental health care, or management of mental illness	n/a	Review article	There is limited research available on mental health in Austronesia<br><br>Many Austronesian-speaking people have experienced recent or current foreign occupation, lack of recognized sovereignty, poverty and low socioeconomic status, and low availability of psychiatric resources and providers.<br><br>There may be relatively unique stressors (e.g. loss of homeland from either global warming or nuclear contamination) affecting people in this region and certain	Solutions to mental health challenges in this world region may include culturally relevant and integrative mental healthcare delivery models; resource preserving, prevention-focused universal mental healthcare; and technology to improve connectivity and increase access to either direct services or workforce-building education and training

				biological profiles (e.g. susceptibility to obesity and metabolic syndrome) that may impact psychiatric treatment.	
Gunasiri (2022)<br><br>[98]	Explore mental health promotion issues related to climate change for young people in Australia since they are highly vulnerable to mental health sequelae	Young adults in Australia	Mixed methods	In this exploratory mixed-method approach, with the engagement of young people living in Australia semi-structured interviews (N = 14) and an online survey (N = 46) were conducted. Thematic analysis and quantitative descriptive statistics were analyzed. Findings indicated that negative impacts included worry, eco-anxiety, stress, hopelessness/powerlessness and feelings of powerlessness/lack of voice.	Climate change was found to be a significant concern for young people and had deleterious effects on mental health. Public health interventions aimed at increased awareness of climate change-related mental health issues may be beneficial and the promotion of nature-based interventions, climate action and empowering social media engagement may be instrumental in limiting mental health sequelae.
Haase (2019)<br><br>[60]	Addresses the relationship between air pollution and health	Adults	Expert analysis	Impacts exist across the life span related to carbon air pollution, particularly for children and older adults with dementia	Mental health professionals should routinely educate patients about air pollution’s relationship with health and work with patients on strategies to participate in the reduction of air pollution in their homes, their communities, and their country
Hanigan et al. (2012)<br><br>[28]	Examine the concern in Australia that droughts increase suicide in farmers and farm workers	Adult farmers and farm workers in Australia	Quantitative analysis to explore the relationship between drought and suicide, stratified by age, sex, and regional subgroups to explore different potential effects of drought, especially on farmers and farm workers	Increased risk of suicide during drought in rural males aged 10–29 years old and 30–49 years old, but a decreased risk in rural females aged >30 years old.<br><br>Rural males aged 30–49 years old who dies by suicide related to drought represented approximately 9% of total deaths in that group over the entire 38 years of the study 1970–2007).<br><br>Increased risk of suicide associated with drought in rural males aged 10–29 years old, supporting the inference that there are flow-on effects to the broader rural community.	Results are consistent with other studies that indicate a link between suicide and climate change. Need further studies on this topic.
Hanigan (2022)<br><br>[99]	Mental health problems are linked with climate change including droughts with suicide noted as one deleterious outcome, thus modeling of climate	Adult data from New South Wales, Australia	Expert analysis via methods for climate change modeling	Suicide rates attributable to drought related to climate change scenarios were explored. Analyses revealed that drought-related suicides increased among rural men aged 10–29 and 30–49 yrs in all modelled climate change models. Rural males aged over 50 yrs and young rural females (10–29) showed no increased	Climate change-related impacts (increased rural suicide rates) may assist in developing mitigation and adaptation strategies to improve community responses to the effects of climate change.

	change associations was studied.			suicide risk, whereas decreased suicide rates were predicted for rural women of 30–49 and 50-plus years of age, suggesting resilience exists in this population in New South Wales.	
Hansen et al. (2011)<br><br>[29]	Aimed to investigate factors influencing the ability of older persons to adapt to hot conditions, and barriers to adaptation	Key personnel involved in aged care, community services, government sectors, emergency services and policy making in Adelaide, Australia	Qualitative study focus groups and interviews with stakeholders	A broad range of factors underpin the heat-susceptibility of the aged<br><br>Psychological issues include fears and anxieties about extreme heat<br><br>Extreme heat can cause distress to older persons<br><br>Those caring for the elderly used terms such as “overwhelming”, “panic”, “anxious”, “concern”, “worried”, “desperate” and “fear” to describe emotional responses to extreme heat<br><br>Stakeholders indicated that during the intense 2009 heatwave, water restrictions, bushfires and extensive media coverage added to the anxiety	Behavioral adaptations during extreme heat are necessary for older persons whose thermoregulatory abilities may be compromised due to age or disease. This investigation of age-related heat-susceptibility and barriers to adaptation has highlighted several issues which may potentially be addressed with public health interventions or policies
Harper et al. (2015)<br><br>[35]	Explore via methods to identify, characterize, and rank climate-sensitive health priorities in Nunatsiavut, Labrador, Canada.	Adults in Nunatsiavut, Labrador, Canada	Mixed methods	This study provided baseline understanding of the climate-sensitive health issues affecting Labrador Inuit, which included mental health and wellbeing among others<br><br>Participants described the environment as being inextricably linked to several determinants of Inuit health, which increases sensitivity to climatic change related impacts on health	Providers should assess for impacts of climate change on affected Inuit populations while recognizing human health and wellbeing are a function of complex social and ecological interactions
Hart et al. (2011)<br><br>[64]	A Rural Adversity Mental Health Program sought to understand the mental health needs of rural families facing drought	Adults in rural families in New South Wales, Australia	Discussion paper	Severe and prolonged drought undermined the economic viability of rural and remote NSW communities and, thereby, exerted pressure on their social fabric, as it did around the country<br><br>Consistent with the well-known indirect harm of financial, environmental and social stressors on mental health, so too	Stakeholders should 1) Identify the emerging mental health needs of rural families including young people, women and older farmers due to drought and environmental events; 2) Develop, and where possible, implement and evaluate programs to address these needs; 3) Identify drought-related mental health needs of Aboriginal communities and any potential pilot

				did drought compromise the mental health of populations in rural NSW<br><br>Mental health and emotional well-being are directly connected to land<br><br>Mental health and well-being suffer due to climate and environmental changes	programs to address these; 4) Improve early intervention strategies and pathways to mental health and drug and alcohol services for rural communities; 5) Improve mental health literacy and reduce the stigma associated with mental health problems; 6) Increase the participation of general practitioners in the improvement of mental health related networks and services designed to assist rural communities adapt to drought and environmental change, and 7) Enhance clinical leadership in the development of mental health-related outreach programs to drought-affected communities; and 8) Create a long-term mental health strategy to assist the adaptation of rural and remote communities to drought and environmental adversity
Hayes (2020)<br><br>[100]	Examine the psychosocial consequences of potential climate change-related major event (public health responses e.g., programs, policies, and practices) with a goal of fostering mental health.	Adults: residents who experienced the 2013 Southern Alberta flood in High River, Alberta	Qualitative	Via descriptive thematic analysis, the findings of this study suggest that there are long-term psychosocial impacts of extreme weather and climate change; unintended consequences related to psychosocial interventions lead to complex emotions and impact psychosocial recovery; and there are perceptions of mental health care, among people exposed to climate-related trauma that occur.	require sustained recovery interventions rooted in local knowledge and interdisciplinary action; and psychosocial recovery is complex after climate-change related events. Public health interventions may be instrumental in guiding climate change and mental health response and recovery interventions.
Hickman (2021)<br><br>[101]	Explore the feelings, thoughts, and functional impacts associated with climate change among young people globally.	Young Adults (Australia, Brazil, Finland, France, India, Nigeria Philippines, Portugal, UK, USA). 1000 participants from each country	Quantitative	Global survey of 10,000 children and young people in 10 countries. data collected on participants thoughts and feelings about climate change and government responses to climate change. Respondents across all countries reported a large amount of worry related to climate change, nearly half of respondents said their feelings about climate change negatively affected their daily lives. countries expressing more worry and a greater impact on functioning tended to be poorer, and in the global south, and more directly impacted by climate change. many respondents reported negative	high levels of distress, functional impact, and feelings of betrayal will negatively affect the mental health of children and young people. recognizing climate anxiety and realities of climate change alongside governmental failures to act are both long term, chronic, and potentially inescapable stressors. increase protection against MH problems by psychosocial resources, coping skills and agency to address and mitigate stressors. Understanding climate change and climate crisis as a human rights issue

				emotions.75% of respondents said the future was frightening. Participants tended to rate government responses to climate change negatively with more feelings of betrayal than of reassurance. negative thoughts, worry about climate change, and impact on functioning were all positively correlated and showed correlations with feelings of betrayal and negative beliefs about government response<br><br>children and young people around the world report climate anxiety and other distressing emotions and thoughts about climate change that impact their daily lives. This distress was associated with beliefs about inadequate governmental response and feelings of betrayal	
Hines-Martin (2021)<br><br>[102]	Examine involuntary displacement of individuals and the impact on mental health (limited to no discussion directly on climate change)	Adults	Commentary	changes to perceived identity and connectedness has had limited attention in literature<br><br>article overall not focused directly on climate change (more broadly community changes and involuntary displacement)	implications for health professionals to utilize expertise with a focus on mental health promotion, poor mental health prevention, and early intervention for this population within a variety of settings
Hollifield et al. (2011)<br><br>[52]	Explicate the term "environmental refugees" and their vulnerabilities related to mental health as well as other health problems	Adult refugees globally	Expert analysis	For climate refugees, mental health effects of climate change events are less well understood than physical health effects. It is known that mental health outcomes differ by the type and severity of events experienced and the social, cultural, and historical contexts in which they occur<br><br>Climate change will affect mental health by direct and indirect pathways	With alienation and a lack of belonging, refugees are prone to demoralization with or without psychiatric disturbances. One study showed that refugees and migrants are often diagnosed with major depression, yet on the average do not benefit from a normal course of treatment<br><br>Climate change will also create a new kind of refugee who will be either nondisplaced or minimally displaced geographically, but who instead will be distressed by a new environment that has been displaced onto them
Howard (2020)<br><br>[103]	Examine the association between climate change risk perception and	Adults: Farmers andranchers in Montana and over 18 years old.	Quantitative	Cross sectional study design through survey administration (written and online), descriptive statistics and correlational tests conducted to evaluate	Maximize public health preparedness efforts, interventions are warranted to provide climate adaptation education and therapeutic outreach specific to agricultural workers

	mental wellbeing among farmers and ranchers in Montana. secondary aim: examine how climate change may be affecting the mental wellbeing of farmers and ranchers in Montana			if climate risk perception was related to levels of mental distress. Open ended survey questions and three scales (including PHQ9 and GAD7) were utilized. Climate change is generating anxiety and distress for farmers and ranchers.	experiencing economic struggle in context of climate change. these findings can inform evidence-based programs to mitigate MH risk of climate change
Hunter (2009)<br><br>[50]	Considers the short, intermediate and long term effects of climate change in relation to the mental health of Indigenous residents of northern Australia, and what these effects mean in terms of supporting adaptation and resilience	Adults: Aboriginal and Torres Strait Islander Australians	Expert analysis	Short term mental health consequences of climate change include with mood impacts, solastalgia, concern, and anxiety. Long term consequences are likely also to be experienced	Providers should work to understanding and mitigate the effects of climate among an already vulnerable population
Hutchins et al. (2018)<br><br>[65]	Examine current governmental public health responses and their future opportunities to improve and protect PMVs (populations with multiple vulnerabilities) from climate impacts through adaptive action	n/a	Expert analysis	PMVs have disproportionately high rates of pre-existing mental illnesses that are exacerbated by climate change<br><br>PMVs experience increased mental illnesses or exacerbation of underlying mental illnesses with injury and premature death<br><br>Climate change threatens to exacerbate health disparities, if needs of PMVs are not central to adaptation planning	A dual adaption plan for public health agencies with universal and targeted interventions that include health equity councils and local minority health directors and their partners can likely protect PMVs from adverse health impacts of climate change
Kabir (2018)<br><br>[36]	Provides an overview of the deleterious effects of climate change on psychological health of the Hill-Tracts and government actions to mitigate the	Adults: Residents in Chittagong, Cox's Bazar, Rangamati, Bandarban and Khagrachhari, Bangladesh	Case study approach (both primary and secondary sources). Primary data were drawn from 125 individuals (in-depth interviews with semi-structured questionnaire) (75 females, 50 males)	Psychological health outcomes ranging from psychological distress, depression, and anxiety, to increased addictions and suicide rates were documented.	Documentation of strong emotional responses led to four pathways through which climate change wasconsidereed ny participants to be impacting psychological health and wellbeing, including increase in drug and alcohol usage, increased family stress, increase in suicide ideation, magnification of trauma

	adverse psychological health impact		from all ages (10 to over 70 years old). Secondary data were collected from books, journals, office records and other published documents		
Karaliuniene (2022)<br><br>[104]	Explore current knowledge on implications of climate change on mental health of children and suggest recommendations for global and local actions from the perspective of early career mental health clinicians and researchers working across WHO regions in socioeconomically and culturally diverse countries	Adults and children	Perspective piece (international/global perspective)	Three domains of climate change related MH effects in children: direct, indirect, and those related to physical conditions<br><br>climate change impacts both the mental and physical health of children and the need to foster resilience and coping strategies is compelling<br><br>introduced recommendations based on the three domains	Includes recommendations relevant for health professions including: establish community support, screen for mental health conditions in areas affected by climate change, develop telehealth services for early ID and monitoring, promote collaboration, establish family therapy programs in vulnerable areas, develop self help resources, foster resilience and coping strategies, implement policies to mitigate risk of suffering, integrate MH screenig in birthing centers, screen for MH conditions in people with physical injuries following weather events, long term MH monitoring, develop diagnostic and management guidelines for MH conditions after climate catastrophes<br><br>urgent need to include in health professions training curricula "climate psychiatry"
Kessler et al. (2008)<br><br>[39]	Explore post-hurricane mental health effects of Hurricane Katrina	Adults: Persons who were exposed to Hurricane Katrina	Quantitative	Prevalence increased significantly in the CAG for PTSD (20.9 vs 14.9% at baseline), serious mental illness (SMI; 14.0 vs 10.9%), suicidal ideation (6.4 vs 2.8%) and suicide plans (2.5 vs 1.0%)<br><br>Unresolved hurricane-related stresses accounted for large proportions of the inter-temporal increases in SMI (89.2%), PTSD (31.9%) and suicidality (61.6%).<br><br>The prevalence estimates of anxiety-mood disorders both at baseline and in the follow-up survey in the New Orleans Metro sub-sample are considerably higher than those found in previous surveys of mental illness after natural disasters in the United States, while the prevalence estimates in the remainder of the sample are comparable to those in	Screening scales are inevitably less precise than clinical interviews, generally leading to associations being attenuated<br><br>Pre-hurricane history of psychopathology that influenced stressor exposure and post-hurricane mental illness may be a factor

				previous studies	
Kousky (2016)<br><br>[45]	Examine: 1) Do disasters have a disproportional effect on children? 2) If so, what are those effects? 3) How long do the effects last? and 4) What can be done to mitigate the harm disasters do?	Children	Expert analysis	Disasters can damage children's physical health. Children may be injured or killed, but they may also suffer from such things as malnutrition caused by disruptions in food supply or diarrheal illness caused by contaminated water. Disasters can cut off access to medical care, even for non-disaster-related illnesses<br><br>Disasters can cause mental health problems. Not only are disasters themselves stressful and frightening, but children can suffer psychological harm from the damage to their homes and possessions; from migration; from the grief of losing loved ones; from seeing parents or caregivers undergo stress; from neglect and abuse; and from breakdowns in social networks, neighborhoods, and local economies<br><br>Disasters can interrupt children's education by displacing families, destroying schools, and pushing children into the labor force to help their families make ends meet in straitened times	More research is needed, especially related to policy and advocacy efforts related to climate change related disasters
Lebel (2022)<br><br>[105]	Examine scientific literature on the effects of climate change on the mental health of Circumpolar Indigenous Peoples; explore how these populations are reacting to and coping with these effects; comment on their implications for clinical practice	Adult: Canadian Inuit	Systematic review	Immediate impacts of climate change on mental health were felt through restricted mobility and disrupted livelihoods. MH effects were further felt through changes in culture and identity, food insecurity, interpersonal stress and conflicts, and housing problems.<br>26 articles met inclusion criteria: 16 focused on Canadian Inuit communities and 21 were exclusively qualitative specifically focused on Indigenous mental health in Circumpolar North.	Understanding climate related pathways of MH risk in the Arctic is crucial to better identify vulnerable groups and to foster resilience. clinicians can recognize and support identified patients, and advocate for policies
Leffers (2022)<br><br>[106]	Examine the impacts and potential impacts of climate change on	Children	Discussion paper	Extreme weather events leave children and families at increased risk for mental health impacts such as anxiety, distress,	Encouraging resiliency, involvement in conservation efforts can help decrease pessimistic outlook. essential knowledge of

	children's health and recommend advocacy, mitigation, adaptation, and resilience strategies to reduce the threats to children's health			depression, fear	climate impacts into plan of care to improve the health of children and families. encourage active transportation, prescriptive time in nature
Ma (2022)<br><br>[107]	Examine the breadth of the literature available and map the key characteristics of previous studies exploring the risk and protective factors that affect the young people's likelihood of experiencing mental health impacts arising from climate change<br>study questions: what is the current landscape of research that explores the risk and protective factors experienced by young people concerning the potential mental health impacts of climate change? how can understanding these risks and protective factors in the context of a socio-ecological system help to inform policy and practice that aim to mitigate the mental health impacts of climate change on young people	Young adults	Scoping review	Evidence base predominantly focuses on young people's experience of PTSD, depression, or anxiety and mostly addresses following severe climate event. discussed risk and protective factors through young people's individual, micro-, exo-, and macro-system levels.<br>92 studies included<br>inclusion criteria included: climate change, mental health and wellbeing, young people, and risk and protective factors.	Awareness of levels of risk and protective factors from individual to macro system level. acknowledging cultural identity as a protective factor against MH impacts of climate change. more research recommended and needed to better understand risk and protective factors, particularly how to promote protective factors against long-term impacts of climate change.

Martin (2020)<br><br>[108]	Describe literature on the impact of the overarching awareness and concerns of climate change on children's mental health and wellbeing. To summarize and clarify terminology related to children's mental health and climate change.	Children	Scoping review	All research that addressed school aged children (3-19 years) mental health issues stemming from an awareness of climate change. did not include direct impacts of climate change on children's mental health such as trauma from specific climate events.	Scoping review protocol. conducted search in 8 research databases, 3 unpublished/grey literature bases. conducted in accordance with JBI methodology. Significant impacts on children's mental health and wellbeing may occur related to climate change with impacts related to climate events.
Martin (2022)<br><br>[109]	Examine the existing literature on the impact of the overarching awareness and concerns of climate change on children's mental health and wellbeing. To summarize and clarify terminology related to children's mental health and climate change.	Children	Scoping review.	Conducted search in 8 research databases, 3 unpublished/grey literature bases with 33 articles included. conducted in accordance with JBI methodology. All research that addressed school aged children's (3-19 years) mental health issues stemming from an awareness of climate change. did not include direct impacts of climate change on children's mental health such as trauma from specific climate events	Mental well being impacts and negative emotions that stem from climate change awareness may be common among child populations, some literature suggests coping through sense of hope and optimism particularly for children
Marzouk (2022)<br><br>[110]	Examine internally displaced persons' perceptions of climate change and its underlying reasons, the importance of addressing climate change and the impacts of extreme hot and cold weather conditions on mental health and psychosocial wellbeing	Adults: Iraqi adult men and women	Quantitative	Iraqi individuals reported on mental health impacts of climate change with extreme heat and psychosocial wellbeing impacted. Irritability was markedly increased during extreme cold weather conditions.	Awareness of resiliency and advocacy efforts in a climate-changing world is critical.
Matthews et al. (2019)	Examine long-term impacts of climate	Adults living in Northern New South	Quantitative Cross-sectional survey	Elevated psychological morbidity among survey respondents 6 months after the	First responders must be able to assess and react appropriately to multiple or high-risk

[51]	change, particularly in communities prone to extreme weather events	Wales		2017 severe flooding in Northern NSW with greater impact on marginalized respondent groups<br><br>Rates of still being distressed about the flood, probable PTSD, anxiety, and depression, and suicidal ideation were particularly elevated in response to three types of exposure: those whose homes or businesses were flooded; those who faced multiple exposures; and those who endured lengthy displacement<br><br>Respondents already experiencing socio-economic marginalization were more likely to be exposed and, if exposed, to have elevated risk of psychological morbidity (i.e., after accounting for extent of flood damage).	exposures<br><br>Care pathways that are individually tailored and sensitive to specific exposures and risk factors may be more effective in preventing the onset of symptoms and in promoting recovery<br><br>The focus of disaster recovery programs needs to be extended beyond the immediate aftermath given research has shown that mental health problems persist for many years
Middleton (2020)<br><br>[111]	Examine personal and collective significance of climate, including changing weather and seasonal patterns, among Nunatsiavut Inuit; and characterize how weather, season, and climate influence mental wellness in the context of climate change	Adults: Nunatsiavut Inuit<br>data drawn from the Inuit Mental Health and Adaptation to Climate Change project	Qualitative	Via semi-structured interviews examine weather impacted mental wellness through three key pathways: shaping daily lived experiences including connection to place and other determinants of wellbeing; altering mood and emotion on a transient basis; seasonally influencing individual and community health and wellbeing	Understanding climate-mental wellness relationship points in multiple pathways is important for action on climate adaptation policy and programming, and underscores the need for more culturally-specific and place-based investigations to appropratiely respond to MH impacts of climate change
Middleton (2021)<br><br>[112]	Examine temporal trends of mental health related clinic visits for the region; examine associations between daily mental health related clinic visits and air temperature variables across the Nunatsiavut region explore whether	Adults: Nunatsiavut (Labrador, Canada)	Quantitative	Via retrospective chart review, incidence rate of mental health related community care clinic visits was greater after two weeks of warm average temperatures and incidence rate of mental health related clinic visits was lower when there was an increase in the number of consecutive days with temperatures considered to be critical to land use.	place-based approaches to health policy, planning, adaptation, and research related to climate change is important particularly in circumpolar regions such as Nunatsiavut

	place quantitatively matters at the climate- mental health nexus				
Morganstein & Ursano (2020)<br><br>[113]	Ecological disasters in a changing global climate have impacts on mental health. Disasters often have a predictable pattern, evolve over time, and lead to psychological and behavioral issues and community disruptions. Various factors enhance transmission of these adverse effects beyond the geographic location of the ecological disaster, with certain populations being particularly vulnerable to these effects.	Adult/family communities	Expert analysis	Psychological and behavioral responses are complex public health burdens following a disaster. A model was developed to understand community phases of recovery post-disaster that include phases of pre-disaster, impact, heroic, honeymoon, disillusionment, and reconstruction.	A model for understanding community responses as well as the cultural and contextual factors that influence their development are critical for effective response and recovery efforts. "Interventions should be optimized for timing and resourcing of recovery efforts. Interventions should be evidence-based, tailored to community needs, and serve to enhance the essential elements of safety, calming, self- and community-efficacy, social connectedness, and hope or optimism. Risk and crisis communication can shape community behaviors and influence perception of risk with trust and health-promoting behaviors being heavily influenced by thoughtful public health messaging."
Nazeer (2020)<br><br>[114]	Changing patterns in climate in the Middle East elicit psychosocial consequences related to climate change disasters which are explored in this analysis. Migration and acculturation stress are also explored.	Children and adolescents: Child and adolescent mental health amidst disasters in Asia, Middle East, and North Africa	Expert Analysis	Disruptions in the human and environment relationship are occurring due to climate change. In order to maintain emotional health, it is essential to address this intersection. Individuals with preexisting mental health problems are more likely to experience decreased physical and emotional health related to climate change. Climate change can indirectly affect individuals by creating psychosocial insecurity, which is also associated with ecoanxiety due to the changing patterns in weather and climate. Extended drought-like conditions lead to complex health challenges and air quality issues, pollen, water-borne diseases, and food and water insecurity. These changing patterns are associated with poor physical and emotional health, as	Child and adolescent mental health is significantly impacted post disaster and must be addressed by clinicians.

				well as psychological disorders and yield increased emergency department visits.	
Nomura et al. (2019)<br><br>[62]	Examines what dimensions of infant temperament were associated with prenatal maternal depression amid Hurricane Sandy exposure	Adults: Mother-child dyads with mothers as respondents	Quantitative	Longitudinal quantitative study measuring prenatal maternal depression (EPDS); Sandy exposure status; and Infant temperament (IBQ-R) with 408 mother-child dyads who experienced Superstorm Sandy.	Monitoring pregnant women at risk of natural disaster/extreme weather event to provide earlier mental health access to mitigate any detrimental effects on infant
Nuvey et al. (2020)<br><br>[115]	Examine struggles of farmers in Ghana in livestock production struggles to meet needs of the people due to population growth and adverse effects of climate change.	Adults: Farmers	Mixed methods	Substantial losses (about 85% n=240/287) of the livestock farmers lost cattle within 1 year. Animal diseases were a major source of loss, as well as theft, and also pasture shortages. Qualitative findings suggested that challenges occurred due to poor access to veterinary services, Results suggested that 60% of livestock farmers had poor mental health with depression (72%), anxiety (66%) and stress disorder (59%) found to be common. Mental wellbeing was negatively associated with the number of adverse events experienced, numbers of lost livestock, emotional attachment of owner with livestock, and self-reported physical illnesses of farmers.	Ghana livestock farmers experience multiple stressful events, with animal health, theft, and pasture shortages associated with climate change yielding mental health consequences for livestock farmers.
Ojala (2022)<br><br>[116]	Examine Sciberras and Fernando's (2021) approach based on an 8-year longitudinal study. Trajectories of climate change worry/anxiety through adolescence and relationships with engagement with news and politics in late adolescence were explored in the original study.	Adolescents	Expert analysis/commentary	Although research has found diverse results regarding climate anxiety as an adaptive or maladaptive response, and whether climate anxiety is adaptive or not, responses to climate anxiety should be considered. Longitudinal studies to examine climate anxiety are needed.	Future examination should address coping with climate change worry and ecoanxiety and consider qualitative approaches.

Olson & Metz (2022)<br><br>[117]	Pregnant mothers, their fetuses, and their children are among those at greatest risk in every population and every jurisdiction. A timely consideration is the health of racialized groups who are particularly vulnerable owing to the confluence of several risk factors that are compounded by climate change.	Adults and children: Pregnant women and children	Expert analysis	Climate change amplifies health inequities for the most at-risk populations. Health challenges also increase allostatic load of those at risk. Allostatic load increases risks to individual and socio-ecological resilience. In the realm of mental health, depression and anxiety and, in the case of pregnant women are increasing. In women and children, the risks are also increasing; for pregnant women, more adverse pregnancy outcomes and impaired development for their newborn children.	New and adapted existing tools are needed to predict challenges for individuals, communities, and populations experiencing climate-related hazards aimed at stress reduction and increasing resilience in populations--particularly pregnant and post-partum women, and their young children.
Pailler & Tsaneva (2018)<br><br>[59]	Examine whether extreme temperature and precipitation in India have an effect on self-reported depression symptoms, cognitive and sleep difficulties, and ability to cope with and control life	Adults: residents of India	Quantitative	Data used from World Health Survey (8468 individuals) and SAGE (7759 individuals) was matched to daily/monthly weather reports. The number of months with warmer than usual temperatures than the previous calendar year have a significant negative effect on psychological well-being increasing cognitive and sleep difficulties, as well as feelings of being unable to cope with or control the important things in life<br><br>Mechanisms through which climate may affect psychological well-being include income shocks and consumption shocks<br><br>Higher temperatures have a significant deleterious effect on stress and lack of agency among individuals in rural, but not urban, areas	Understanding the effects of climate on psychosocial well-being and identifying potential resolutions is critical for insuring vulnerable populations.
Palinkas & Wong (2020)<br><br>[13]	Examine mental health impacts of three types of climate-related events: (1) acute events such as hurricanes, floods, and wildfires; (2)	Adults	Expert analysis	The paper describes results from both direct (i.e. heat stress) and indirect (i.e. economic loss, threats to health and well-being, displacement and forced migration, collective violence and civil conflict, as well as environmental changes) as uniquely adding to psychological distress and climate	The most profound mental health outcome related to global climate change is the associated the existential threat that occurs with direct and indirect effects of climate change.

	subacute or long-term changes such as drought and heat stress; and (3) the existential threat of long lasting changes in climate, particularly related to heat stress, rising sea level, and changes in land leading to climate anxiety. Climate impacts are explored as both direct (i.e. heat stress) and indirect (i.e. economic loss, threats to health and well-being, displacement and forced migration, collective violence and civil conflict as examples.			anxiety.	
Parry et al. (2019)<br><br>[71]	Examine health risks particularly for vulnerable populations	Adults and families	Discussion paper/Conceptual framework proposal	This paper focuses on knowledge gaps concerning health risks from climate change are unjust, and must be addressed, because they hinder evidence-led interventions to protect vulnerable populations.	Risks of climate change may be invisible and disproportionately address vulnerable populations.
Patel et al. (2021)<br><br>[118]	Examine adverse effects of climate change on children. To explore the fact that mental health impacts may not be immediately evident and that a risk exists that children who experience climate-related trauma or toxic stress may not receive appropriate treatment and care	ChildrenWith adults surveyed	Discussion paper	Literature analysis in three key areas with 3 key subjects: ecoanxiety, climate change and natural disasters as adverse childhood experiences, and displacement after an environmental disaster. Survey responses from 7 key informants. Eco-anxiety is a rapidly emerging mental health consequence of climate change in children and youth. Emerging literature suggests that children and youth have increasing worry about planetary health. Ecoanxiety may emerge and manifest from directly experiencing or observing climate related events such as wildfires.	Implications include the need for addressing gaps in the literature related to the intersection of racism and climate change. Although impacts of climate change on communities of color are examined in the literature, more research and scholarship is needed about social determinants of health as an indicator.

	and carry mental health impacts into adulthood.			Persistent mental health outcomes may occur and increase as climate events increase. As a novel term, eco-anxiety is important to understand related to children's mental health, trauma, and stress.	
Patrick et al. (2022)<br><br>[119]	Examine the impact of climate change on mental health in the Australian population and identify populations who are most at risk of climate-related mental health.	Adults	Quantitative/Survey	Most Australians respondents reported having a direct experience of a climate change–related event. Young people reported experiencing significant rates of eco-anxiety—a mental health consequence. Twenty five percent of respondents with direct experience of a climate change–related event met post-traumatic stress disorder screening criteria. However, respondents who did not report a direct experience demonstrated symptoms of pre-trauma, particularly in younger age groups and women. In this sample, 9.4% reported significant eco-anxiety, 15.7 reported pre-traumatic stress, and 25.6% reported post-traumatic stress disorder.<br><br>The population was aimed at collecting data via a nationwide Australian survey conducted between August and November 2020. The populations was representative of the general population across sex, age, location, state and area, and sociodemographics. Two-stage recruitment involved unrestricted self-selected community sample through mainstream and social media ($N = 4428$) and purposeful sampling using an online panel ($N = 1055$)."	In Australia, where climate change is an emerging challenge, mental health issues are pervasive. Those with and without direct experience of climate change are reporting significant mental health impacts, with younger age groups being disproportionately affected. It is critical for health care professionals to address the emerging mental health challenges and limit climate-related mental health burdens.
Paxson et al. (2012)<br><br>[120]	Examine the long-term effects of the hurricane on the mental health for those who experienced Hurricane Katrina with a sample of low-income mothers.	Adults: Low income women	Quantitative/longitudinal survey design	The results of this longitudinal survey suggest that post-traumatic stress symptoms (PTSS), and symptoms of psychological distress (PD), exist five years post Hurricane Katrina in this sample of low-income mothers. Although PTSS has declined over time after the hurricane, it remained high nearly four to five years later. PD levels also declined,	Among low-income mothers who were Hurricane Katrina survivors, mental health issues including post-traumatic stress symptoms either alone or with co-morbid psychological distress were reported and require urgent attention from health care providers. For those who experienced hurricane-related trauma and had poor mental health or low socioeconomic status prior to

				but did not return to pre-hurricane levels. At both measurement points psychological distress before the hurricane, hurricane-related home damage, and exposure to traumatic events were associated with PTSS that co-occurred with PD.	the hurricane, mental health issues were further challenging post-hurricane.
Pennea (2021)<br><br>[121]	This paper explores the nexus of climate change, COVID-19, and issues of environmental justice.	Children: Impacted by climate change and COVID-19	Discussion paper	All children are at risk from the effects of climate change and COVID-19, however this paper addressed the disproportionate impact on children in marginalized poor communities and communities of color.	Children are facing greater negative health impacts from both climate change and COVID-19 as public health crises. National strategies for addressing these concerns in marginalized, at-risk populations.
Petrasek et al. (2015)<br><br>[30]	Identifies and characterizes youth-identified protective factors that enhance mental health and well-being, focusing on factors that are challenged by climatic and environmental change	NunatsiavutInuit Settlement	In-depth semi-structured qualitative interviews with 17 youth (ages 15-25)<br><br>Constant comparative method used to continuously compare emerging concepts and codes between and within interviews	Indigenous communities will face more serious challenges from climate change given their often-high dependence on the natural environment<br><br>Climate change is related to worry, anger, frustration, anxiety, stress, feelings of helplessness	It is important to understand the characteristics, opportunities, and resources of individuals, families, and communities that protect and promote youth mental health and well-being<br><br>Social and community connectedness can improve resiliency and coping strategies for vulnerable populations
Picou (2010)<br><br>[122]	Examination of whether the September 2013 floods were associated with local climate change beliefs and actions.	Adults: Boulder Colorado residents	Case study analysis	Climate change events did not alter individuals' climate change beliefs, however individuals experienced a greater sense of vulnerability to and increased awareness of the risks posed by climate change. Residents and policymakers placed greater emphasis on adaptation to future extreme events.	Resilience through social capital may impact climate change beliefs with a differential impact in vulnerable and marginalized populations.
Polain et al. (2011)<br><br>[123]	Describe the circumstances and mental health of older farmers who experienced prolonged drought in NSW; and report policy and service approaches that might support these farmers	Adults: Farmers	Qualitative	Farmers in rural Australia experience loss which contributes to mental health sequelae as a result of prolonged drought from climate change	Addressing healthcare needs of vulnerable populations is critical before the next extreme weather event (or drought)

Pollack et al. (2016)<br><br>[53]	Examines the mental health and life functioning, and their predictors, of people living in central coastal Vietnam—an area characterized by high risk for natural disasters and poverty	Adults in coastal central Vietnam	Quantitative	Studying populations exposed to frequent natural disasters in the face of global climate change<br><br>Individuals with exposure to traumatic major storm was predictive of any mental or self-perceived physical health problems, but frequency of major storm exposure was not a significant predictor<br><br>Global climate change potentially places communities at significantly elevated risk for mental health problems through increased traumatic exposure and through indirect effects of less severe storm exposure	Disaster risk reduction and response efforts should consider pre-disaster community factors, such as economic conditions, in responding to and assessing the likely impact of disasters on community mental health and functioning
Reacher et al. (2004)<br><br>[58]	Investigate new episodes of illness and psychological distress in adults following severe river flooding	Adults	Quantitative	Psychological distress was found to be a leading adverse health outcome associated with flooding and may explain a proportion of physical illness	Awareness and screening of psychological distress following extreme weather event
Rigby et al. (2011)<br><br>[54]	To summarize the ways in which Aboriginal people believe prolonged drought might impact their social and emotional well-being in rural and remote NSW, as well as possible adaptive strategies people proposed or had tried	Adults: Aboriginal people in New South Wales	Qualitative	Findings indicated that participants experienced complex issues related to social and emotional well-being. Drought contributed to shame, despair, aggravating socioeconomic disadvantage	Providers should be aware of mental health sequalae from extreme weather events.
Rygaard (2021)<br><br>[124]	Examine the impacts on mental health of European families and children and relationship with massive migration and urbanization with industrialization, overpopulation, and long-term effects of	Children and families: European children and families	Discussion paper	Blended online learning programs were developed to address the issues of mental health in families impacted by migration related to climate change.	Mental health risk factors, climate change, and migration/change in living circumstances may weaken protective family networks and cause elevated parental stress in a climate-changing world.

	climate change.				
Saniotis & Irvine (2010)<br><br>[77]	Examine two issues in relation to climate change and older Australians: first, pharmacology and autoregulation; and second, mental health among older Australians	Adults: Older Australian adults	Expert analysis	Farmers in rural and remote areas are vulnerable to mental illness due to isolation and exposure to environmental hazards, including increased rates of suicide. Financial losses from extreme weather events can precipitate depression, anxiety, family break down, grief, anger. Climate change may have potential direct or indirect health impacts on older Australians, particularly related to pharmacology and thermoregulation and mental health. Extreme heat and weather events may lead to long-term disruption to rural infrastructure and social networks, thus reduced coping	More research is needed on the impact on mental health of older Australians and climate change in the following areas: climate change and social communication networks; and mental health of older people in the remote and rural sectors<br><br>Social and public health sciences are important in demonstrating the role between climate change and addressing possible uncertainties at rural community levels
Schramm (2020)<br><br>[125]	Examination of two tribes—the Pala Band of Mission Indians and the Swinomish Indian Tribal Community—and implementation of the Climate-Ready Tribes Initiative.	Adults: Members of the Pala Band of Mission Indians and the Swinomish Indian Tribal community	Discussion paper/expert analysis	The adaptation capacity building and technical needs in tribes were explored in this collaborative Centers for Disease Control and Prevention (CDC) and National Indian Health Board (NIHB) Climate-Ready Tribes Initiative (CRTI) in 2016. Each tribe experiences specific climate hazards and sensitivities, in this analysis, Pala Band utilized the Tribal Psychosocial Climate Resilience Framework and the Swinomish's utilized the I-BRACE framework developed by the CDC. These approaches were designed to help tribal communities incorporate their own data and traditional knowledges into climate change adaptation work.	As noted by the authors, there are lessons learned from the ClimateReady Tribes Initiative that may inform climate and health policy and practices nationwide.
Sciberras & Fernando (2022)<br><br>[126]	Examine longitudinally whether Australian adolescents' climate change-related worry is associated with the presence of depressive symptoms and increased political engagement	Adolescents: Australian adolescents	Quantitative	13% of adolescents reported high persistent worry. 24.9% reported moderate worry. 24.3% reported increasing worry. 16.8% reported persistent low worry. Adolescents with high persistent worry had higher depressive symptoms at age 18–19 years. The high persistent and increasing worry groups reported more engagement with news and politics	Health professionals can validate and help adolescents manage their worries about climate change and support climate activism. Providers may also support parents to have meaningful conversations with their children about climate change. Encouragement from providers to discuss climate-related worries may help young people develop more adaptive coping strategies
Sharpe &	Conduct a scoping	Children	Scoping review	All studies assessed the association	Climate-related disaster exposure severity

Davison (2022 [127].	literature review to determine how exposure to climate change and climate-related disasters influences the presence of mental disorders among children in low- and middle-income countries			between a climate-related disaster exposure and a mental disorder outcome, while none explored broader climate change-related exposures. Post-traumatic stress disorder (n = 21 studies) and depression (n = 8 studies) were the most common mental disorder outcomes.	(i.e., level of exposure) is a risk factor for mental disorders, with significant exposure-outcome associations identified. Cognitive behavioral therapy, narrative exposure, meditation relaxation, group therapy, and play therapy may be used to improve mental health. Disaster relief workers should be trained to support children in these situations, such as through the use of emotional first aid
Shultz et al. (2019) [66]	Examine the theme of environmentally forced migration and the effects of population displacement on mental health and psychological functioning using case examples	Adults	Case discussion/discussion paper	Climate change drives environmentally induced forced migration through multiple pathways and sets off cascades of public health and mental health consequences which will require a multifaceted approach to address migration's intersection with climate change.	Public health and mental health responses are needed in a climate-changing world with migration challenges.
Silveira et al. (2021). [128]	Examine the mental health sequelae of wildfire in California (the 2018 Camp Fire)	Adults: In California	Quantitative	Direct exposure to large scale fires significantly increased the risk for mental health disorders, particularly for PTSD and depression. Childhood trauma and sleep disturbances exacerbated mental health symptoms. Self-reported resilience had a positive effect on mental health, and mindfulness was associated with significantly lower depression and anxiety symptoms	Those exposed to wildfire should be assessed for pre-existing vulnerabilities, particularly childhood adversities and quality of sleep. Providers should promote resiliency skills (e.g., mindfulness and exercise) as they may be an effective tool in disaster relief efforts and programs
Singh et al. (2022). [129]	To investigate the enabling and inhibiting factors that affect the ability of iTaukei (Indigenous Fijian) women to contribute to building community resilience	Adult Women: In Fiji	Ethnographic qualitative inquiry using semi-structured interviews, focus groups, and participant observations	iTaukei women contribute significantly to three key areas of community resilience (social resilience, economic resilience and ecological resilience). iTaukei women face barriers that prevent them from contributing to decision-making on issues related to climate change resilience in the community	Community providers should promote interventions that are culturally accepted and that also support the role of women in decision-making, while promoting their participation in various climate change forums in the community
Timlin et al. (2021) [130]	Evaluate the impacts of climate change and permafrost thaw on mental wellness in Disko Bay,	Adults: In Greenland	Mixed methods	Thematic analysis revealed four themes: 1) Good everyday life, 2) Living challenges, 3) Changing nature and environment, 4) Uncertain future, and 5) Bright future. The presence of	Four themes related to impacts of climate change on wellbeing may be helpful in addressing mental health in Greenland residents.

	Greenland			respondents' professional activity was significantly associated with self-rated satisfaction with life and quality of life. A significant association was also found between well-being and age. Participants aged 24–35 were most likely to report a very good well-being. Adults aged 45–54 years and 55–64 years were most likely to report not having a very good well-being.	
Torres & Casey (2017)<br><br>[56]	Propose a conceptual framework that illustrates the disruption of social ties is a key mechanism by which climate-related migration may negatively impact mental health	Adults	Commentary	Regarding migration, studies indicate poorer mental health among migrants. Suggested reasons include losing access to natural landscape, increase in traumatic circumstances and disruption in social and community connections. The latter may result in social isolation, reduced social and material support, reduced sense of belonging. Migration related separation may compound the impact of adverse climate change related experiences such as acute weather events, food and water insecurity the loss of home or land and reduced social cohesion as a result of increased competition over basic resources. Evidence suggests that the out migration of family members impacts the mental health negatively of those who are left behind and are compounded by other climate-related vulnerabilities	Collaborations between international organizations, national governments, and the private sector are needed to incorporate technology advances into efforts to re-connect mobile populations and to facilitate social connection. This is particularly important given the protracted nature of migration.
Tschakert et al. (2013)<br><br>[131]	Assess threats to livelihoods and sense of belonging in deteriorating landscapes; and to explore potential manifestations of solastalgia	Adults: Migrants from northern Ghana	Qualitative	The main reasons for migrating, in addition to lack of income, were no access to fertile land and no jobs for existing skill levels. Negative changes in the physical landscapes over several decades (disappearance of shade through trees, loss of beauty, and the drying up of wells, ponds, and dams) triggered acute feelings of sadness. Extreme sadness (depression) emerged as the most frequently elicited emotion	Providers can encourage a critical stance toward real, anticipated, and generational loss that embraces both mourning and a shared feeling of vulnerability.
van Nieuwenhuizen,	Examine the effects of climate change on	Children and adolescents	Commentary/literature review	Climate change is a social and ecological determinant of health for children and	Psychiatry and psychology training programs should integrate climate change content into

et al. (2021)<br><br>[132]	child and adolescent mental health; and to discuss treatment and engagement by clinicians			adolescents. It is also a source of trauma and distress. Single extreme weather events contribute to significant negative mental health consequences; however, sub-acute and chronic climate events also have mental health sequelae. Awareness of the climate crisis is associated with emotional distress. Those with pre-existing mental illness and low social support are at elevated risk for climate change-related mental health effects. Climate activism is associated with resilience and positive development, but may also be a source of increased stress, particularly for marginalized youths	their curricula. Mental health clinicians should assess and treat the sequelae of climate change including depression, anxiety, PTSD, and suicidality. Providers should encourage adaptive coping by normalizing and validating young people's climate-related concerns, encouraging stress reduction, and identifying ways to increase agency and resiliency
Vernberget al. (1996)<br><br>[133]	Examine the emergence of posttraumatic stress disorder symptoms in in children three months after Hurricane Andrew	Children	Quantitative	14% of children reported having few or no symptoms of PTSD related to the hurricane. Approximately 30% of children reported mild, 26% moderate, 25% severe, and 5% very severe symptoms. Lower levels of social support were related to greater PTSD symptomatology.	A conceptual model that includes Traumatic Events, Child Characteristics, Access to Social Support, and Children's Coping may be helpful for future research and intervention efforts in the wake of natural disasters
Wang et al. (2014)<br><br>[134]	To assess the effects of extreme ambient temperature on hospital emergency department visits related to mental and behavioral illnesses	Adults: Emergency Department visits	Quantitative via time-series study	There was a strong association between mental/behavioral visits and mean daily temperature. A 29% increase in mental/behavioral visits was observed over a cumulative period of 7 days after exposure to high ambient temperature. Similar associations were reported for schizophrenia, mood, and neurotic disorders.	Providers should assess for mental health exacerbations during periods of high temperatures. Those with cognitive impairments may be unable to communicate their distress. Medication review should be conducted, as some psychotropic medications disrupt normal thermoregulatory mechanisms.
Xu et al. (2018)<br><br>[61]	Explore the potential relationship between hotter climate and children's mental health in Australia	Children: Australian children	Quantitative	Boys' mental health outcomes were influenced by environmental and locational factors including average daily temperature; girls more by household and socioeconomic factors<br><br>While the direct effect is significant for boys' mental health, it is not for girls. The indirect effect of temperature is significant on girl's mental health due to decreased participation in organized physical activity	Significant for future policy to help mitigate heat-related mental health impacts on children.

				SDQ subscale results indicated that in hotter temperatures, children are more likely to be restless and or easily distracted.	
Ziegler et al. (2019)<br><br>[135]	Examine the impacts of climate change in context of conflict, drought, and famine	Adults; Families	Discussion paper/Clinical guidelines report	Posttraumatic stress disorder, depression, and acculturation stress may result from the devastating impacts that climate change will have on individuals, families, communities, and even entire countries in cases of conflict, drought, or famine	Conducting mental health evaluations and providing trauma-informed counseling for patients experiencing stress from climate-related events and assessing families for food security are critical roles for the primary care provider practicing in the age of climate change
